# DEspR Roles in Tumor Vasculo-Angiogenesis, Invasiveness, CSC-Survival and Anoikis Resistance: A ‘Common Receptor Coordinator’ Paradigm

**DOI:** 10.1371/journal.pone.0085821

**Published:** 2014-01-21

**Authors:** Victoria L. Herrera, Julius L. Decano, Glaiza A. Tan, Ann M. Moran, Khristine A. Pasion, Yuichi Matsubara, Nelson Ruiz-Opazo

**Affiliations:** Department of Medicine and Whitaker Cardiovascular Institute, Boston University School of Medicine, Boston, Massachusetts, United States of America; University of Queensland Diamantina Institute, Australia

## Abstract

*A priori*, a common receptor induced in tumor microvessels, cancer cells and cancer stem-like cells (CSCs) that is involved in tumor angiogenesis, invasiveness, and CSC anoikis resistance and survival, could underlie contemporaneous coordination of these events rather than assume stochasticity. Here we show that functional analysis of the dual endothelin1/VEGFsignal peptide receptor, DEspR, (formerly named *Dear*, Chr.4q31.2) supports the putative common receptor paradigm in pancreatic ductal adenocarcinoma (PDAC) and glioblastoma (GBM) selected for their invasiveness, CD133+CSCs, and polar angiogenic features. Unlike normal tissue, DEspR is detected in PDAC and GBM microvessels, tumor cells, and CSCs isolated from PDAC-Panc1 and GBM-U87 cells. DEspR-inhibition decreased angiogenesis, invasiveness, CSC-survival and anoikis resistance in vitro, and decreased Panc1-CSC and U87-CSC xenograft tumor growth, vasculo-angiogenesis and invasiveness in nude^nu/nu^ rats, suggesting that DEspR activation would coordinate these tumor progression events. As an accessible, cell-surface ‘common receptor coordinator’, DEspR-inhibition defines a novel targeted-therapy paradigm for pancreatic cancer and glioblastoma.

## Introduction

Cancer recurrence and invasiveness remain without effective therapy despite initial therapeutic response of the primary tumor. This is especially true for pancreatic adenoductal carcinoma (PDAC) and glioblastoma multiforme (GBM), both of which have the lowest median survival among cancers despite extensive research. Emerging data implicate cancer stem-like cells (CSCs), or tumor/cancer-initiating cells, which possess stem-like properties of prolonged self-renewal and potential to generate “heterogeneous lineages of cancer cells that comprise the tumor” [1], [2] and are comprised of different immunophenotypes [3]. Although the origin(s) and dynamic heterogeneity of CSCs remain to be elucidated, cumulative studies report innate chemotherapy resistance, survival in adverse microenvironments, anoikis resistance, increased tumorigenicity [4]–[6], proangiogenic [4], [7], [8] and vasculogenic competence of CSCs in different solid tumors [9]–[12], thus suggesting CSCs as logical targets for anti-cancer therapies [13]. However, the complexities of CSC heterogeneity and plasticity present obstacles to CSC-targeted therapy development [14]. To overcome these obstacles, identification and subsequent inhibition of a receptor common to CSCs and tumor vascular cells involved in tumor progression paradigms that would apply regardless of CSC subtype, should provide an alternative tactical targeted therapy approach. Since cancer is in essence aberrant organogenesis [15], recurrent and micrometastatic tumor growth require vascularization to progress [4], [7], [8], [16], [17]. Not surprisingly, normal and pathological vascularization, as well as cancer recurrence and invasiveness all require survival mechanisms in their hypoxic microenvironments, anoikis resistance for cell migration [7], [8], and just like normal stem cells, CSCs localize to and interact with a vascular niche [18]. However, given the absence of vascular endothelial growth factor (VEGF)-receptors on CSCs [7] and non-involvement in anoikis resistance [19], we hypothesize that nonVEGF-receptors comprise the common receptor system involved in tumor progression paradigms, CSC-vascularization interactions [20], [21] and CSC-microvascular niche [22] – such as the dual endothelin-1/VEGF-signal peptide receptor, DEspR [22], localized to human chromosome 4q31.2 (Figure S1A) with gene name, *Dear*, GenBank accession EF212178.

DEspR roles in cancer are deduced from its embryonic-lethal null mutation phenotype resulting in E10.5–12.5 day embryonic lethality characterized by abnormal vasculogenesis with incomplete dorsal aorta formation, and by absent angiogenesis, and failed endocardial-to-mesenchymal transition/migration resulting in thin-walled hearts [22]. The DEspR null mouse phenotype is similar to, but is distinguished from the heterozygous VEGF^+/−^ knockout mouse phenotype [23], and from the homozygous knockout mouse phenotype of its overlapping opposite-strand transcript, Fbxw7, a ubiquitin ligase oncosuppressor [25]–[27] by the detection of hyperconvoluted neuroepithelium throughout the neural tube [22], suggesting a DEspR-specific role in neuroepithelial stem cell-to-radial cell transition and/or migration. Furthermore, 50% reduction of DEspR expression in heterozygous DEspR^+/−^ knockout mice is not embryonic lethal [28] in contrast to the embryonic-lethal phenotype of heterozygous VEGF^+/−^ knockout mice [23], [24], and decreased tumor growth in DEspR^+/-^ male mice [22], in polar contrast to increased tumorigenesis expected from the loss of Fbxw7-tumor suppressor functions as seen in human cancer and mouse tumor inactivating mutations [27], [29]. Importantly, DEspR inhibition at the protein level via an anti-ratDEspR-specific polyclonal antibody decreased tumor growth, tumor vascularization, and nuclear malignancy-grade in irradiation-induced rat mammary tumors [22], thereby clarifying DEspR-specific pro-tumorigenic roles in contrast to the tumor suppressor roles of Fbxw7. Confirmatory immunohistochemistry detected DEspR+ expression not only in rat mammary tumor blood vessels, but also in tumor cells and in invading tumor cells (data not shown). Additionally, DEspR-signaling, studied in human DEspR-positive permanent Cos1-cell transfectants phosphorylates Akt in a dose-response manner [30].

Given these observations, we tested the hypothesis that human DEspR is a common receptor mechanism in human cancer cells, CSCs, and microvessels, which underlies a putative coordinating paradigm for tumor progression events. To test this hypothesis, we used combinatorial in vitro and in vivo experimental systems testing DEspR roles in two human cancers with the lowest 5-year survival rates and polar vascular phenotypes, pancreatic adenoductal carcinoma (PDAC) and glioblastoma multiforme (GBM).

## Results

### DEspR-ligand interactions and ‘common receptor’ expression in tumor microvessels, tumor cells and CSCs

**Figure 1:**
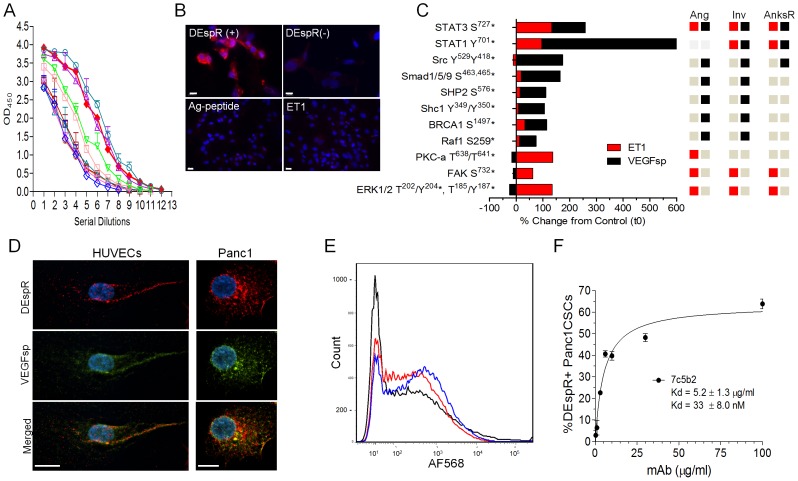
DEspR expression in Cos1 cell transfectants, HUVECs and CSCs. (A) Immunostaining with 7c5b2-mAb detects DEspR expression in Cos1cell DEspR-positive transfectants (DEspR+Cos1), but none in mock-transfected DEspR-negative Cos1cell controls, (DEspR(-)Cos1). Anti-hDEspR 7c5b2-binding on DEspR+Cos1cell-transfectants is displaced by 100X antigenic peptide (Ag-peptide) and by 100X endothelin-1 (ET1). ELISA of 7c5b2 binding to Ag-peptide in Fig. S1B. (B) Bar graph summary of signaling phosphoproteins with greater than 50% change from control (CFC) detected on phosphoproteomic analysis upon VEGFsp-DEspR (black bars) and ET1-DEspR (red bars) activation in DEspR+ Cos1 cell transfectants; see Figure S1C for representative phosphoprotein fluorescent image. Role classification for angiogenesis, invasiveness and anoikis resistance presented based on PubMed publications. Red square, ET1-DEspR activation of phosphoprotein; black square, VEGFsp-DEspR activation of phosphoproteins; gray square, no reports of involvement in said pathway. (C) Confocal photomicroscopy of DEspR (red) and VEGFsp (green) localization and co-localization in endothelial cells (HUVECs) and (D) PDAC Panc1 cells using 7c5b2 anti-hDEspR mAb and anti-VEGFsp polyclonal antibody. DAPI, blue; bar, 10 microns. (E) Representative immunofluorescence images of anchorage-independent PDAC Panc1-CSC and glioblastoma U87-CSC singlets and spheroids after dispersal by mechanical trituration. DEspR+ immunostaining (red); DAPI nuclear stain (blue); bar, 20 microns. Phase contrast images of spheroids ≥50 microns. (F) Superimposed composite of competition FACs analysis of anti-hDEspR 7c5b2 mAb binding to Panc1-CSCs alone (blue), and when displaced by the addition of DEspR ligands at 50×: VEGFsp (black), ET1 (red) respectively. (G) Binding affinity of anti-hDEspR 7c5b2 to Panc1-CSCs showing Kd = 33±8.0 nM.

To advance the study of DEspR in human tumorigenesis without confounding effects due to the partial overlap of DEspR (gene name *Dear*) with Fbxw7 exon-5 on opposite strands (Figure S1A), we developed human-specific anti-DEspR monoclonal antibodies and selected 7c5b2 mAb, with high binding affinity (Figure S1B) using the same antigenic peptide validated for a human-specific anti-DEspR polyclonal-antibody (pAb) [31]. To ascertain binding specificity, we performed 7c5b2 mAb-immunostaining of hDEspR+ permanent Cos1 cell transfectants compared to DEspR-negative, mock-transfected Cos1 cells. As shown in Figure 1A, we detected DEspR+ immunofluorescence only in DEspR+ Cos1 cell transfectants, confirming Western blot results of DEspR+ Cos1 cell permanent transfectants using the anti-hDEspR polyclonal ab [31]. To further confirm 7c5b2 specificity, blocking experiments were done which detected that 7c5b2-immunostaining of DEspR+Cos1 cell transfectants was effectively blocked by the antigenic peptide and by ET1 (Figure 1A), concordant with high affinity binding of ET1 to human DEspR [31], as well as to rat and mouse DEspR [22]. To test functionality of DEspR binding by its ligands, ET1 and VEGFsp, we performed phosphoproteomic analysis of ET1- and VEGFsp-specific DEspR signaling using permanent DEspR+Cos1 cell-transfectants (Figure 1B). This detected differential ligand-specific DEspR-mediated signaling with a few overlaps. As shown in Figure 1B, Figure S1C, and Table S1, DEspR-mediated signaling activated phosphoproteins implicated in angiogenesis with some overlap with VEGF-VEGFR2 signaling pathways (FAK, PKCa, ERK1/2), but also distinct from VEGF-VEGFR2 with the activation of Src Y^259^Y^418^, STAT1 Y^701^, STAT3 S^727^, Smad 1/5/9 S^463,465^ (Figure 1B, Table S1). Collectively, these findings support the functionality of VEGFsp- and ET1-binding to DEspR.

Since the multifunctionality of VEGFsp beyond targeting of the VEGF propeptide to the endoplasmic reticulum is apparent given specific binding of VEGFsp to DEspR [22], [31] and stimulation of VEGFsp-DEspR-specific signaling (Figure 1B), we studied DEspR-VEGFsp co-localization in human umbilical vein endothelial cells (HUVECs) and pancreatic cancer Panc1 cells. Confocal microscopy of double immunostained HUVECs (Figure 1C) and Panc1 cells (Figure 1D) with anti-DEspR mAb-7c5b2 and anti-VEGFsp pAb detected DEspR expression on HUVECs and Panc1 cell membranes. Interestingly, Panc1 cells also localize DEspR in the cytoplasm and in the nuclear membrane and/or perinuclear area (Figure 1D). Merged images show colocalization of VEGFsp and DEspR in HUVECs (Figure 1C) and Panc1 cell membranes (Figure 1D), as well as in the nuclear membrane and perinuclear area of Panc1 cells (Figure 1D).

To analyze DEspR-expression in CSCs representative of multiple CSC-subsets [3], we studied DEspR-expression in anchorage-independent Panc1-derived and U87-derived CSCs which were selected and functionally validated for anoikis resistance, stemness-associated tumorsphere formation in non-adherent conditions through seven passages, septenary CSCs (Figure 1E), and tumorigenicity in outbred nude (*nu/nu*) rat xenograft models. In contrast to zero-tumorigenesis of 2-million non-CSC Panc1 tumor cells in orthotopic (n = 20) and heterotopic (n = 20) locations in nude rats (data not shown), 2-million Panc1- and U87-CSCs produced heterotopic tumors in ∼50–100% of nude rats respectively, without pharmacological immune suppression.

To further explore ligand-specific binding to DEspR, we isolated anchorage independent tumor cells from the Panc1 cell line, Panc1-CSCs. FACS analysis detected successful competition of DEspR ligands, ET1 and VEGFsp thereby blocking, hence reducing, 7c5b2-binding to DEspR+ Panc1-CSCs at 4°C (Figure 1F), while concentration-dependent binding of fluorescently labeled 7c5b2 mAb to Panc1 CSCs was observed giving a Kd = 33 nM (Figure 1G). Altogether, these observations corroborate the specificity of 7c5b2 binding to DEspR. We note that VEGFsp seems to have competed with 7c5b2 binding more effectively than ET1 at 50X.

### Increased DEspR expression in PDAC and GBM tumor blood vessels and cancer cells

**Figure 2:**
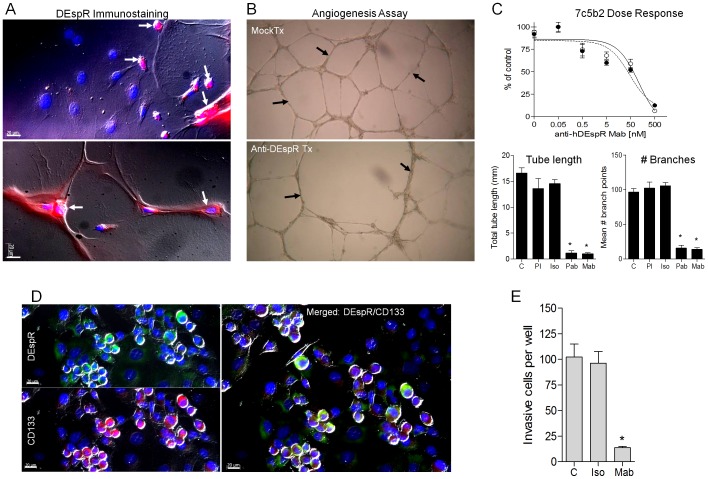
DEspR expression in human pancreatic ductal adenocarcinoma (PDAC) and glioblastoma (GBM). (A) Merged immunofluorescence image of Gr. IV PDAC tumor tissue showing DEspR+ expression (red) in PDAC ductal tumor cells (white arrows) and alpha smooth muscle actin (aSMA)+ expression (green) in adjacent stromal tissue (upper left). DEspR+ and aSMA+ expression and co-expression (yellow) in PDAC tumor microvessels (lower left). Merged immunofluorescence images of normal pancreas detects no DEspR expression in ducts, parenchymal cells (upper right) nor in microvessels (white arrows, lower right). Differential interference contrast (DIC) overlays showing clusters of DEspR+ (red) microvessels (yellow arrows) and co-expressed DEspR+/aSMA+ (yellow) in microvessels (white arrows) within stromal fibrosis area (lower left) in pancreatic cancer but none in normal tissue. (B) Individual and merged immunofluorescence images of DEspR (red), CD133 (green) and double DEspR+/CD133+ immunofluorescence (yellow) shows DEspR+,CD133+ co-expression (yellow) in pancreatic cancer cells at invasive tumor edge (white arrows). Normal pancreatic tissue does not exhibit DEspR+ and CD133+ immunostaining. (C) DEspR+ (red) GBM tumor cells and microvascular endothelium at tumor edge with CD133+ (green) putative CSCs at GBM tumor edge. Merged DEspR+/CD133+ co-expression (yellow-green or yellow) in GBM putative CSCs (white arrows) close to DEspR+ microvessels (yellow arrows). AF568-labeled isotype control (Isotype) shows negative immunofluorescence of GBM section at identical experimental settings; microvessel (yellow arrow). No DEspR+ expression detected in normal brain section. Tumor biopsy core analysis in Figure S2. Bar, 20 microns.

To define a translational framework, we then studied DEspR expression in pancreatic adenoductal carcinoma and glioblastoma, selected for their high unmet need, similar invasiveness but polar vascular phenotypes. Using 7c5b2-AF568 labeled mAb, immunostaining of human tumor tissue sections detected increased DEspR expression in Grade-IV pancreatic adenoductal carcinoma tumor cells and microvessels, even in areas of stromal desmoplasia (Figure 2A), in marked contrast to low or no expression in normal pancreatic parenchymal and ductal cells, and microvessels (Figure 2A). Detection of DEspR expression in both alpha smooth muscle actin (aSMA)-positive and aSMA-negative tumor vessels (Figure 2A) suggests DEspR roles on neovessels and pericyte-end sheathed microvessels. To study DEspR co-expression with CD133, a marker for PDAC CSCs [32], we analyzed CD133/DEspR double immunostaining of tumor biopsy core array. Consistent with the detection of DEspR on anchorage independent Panc1-derived CSCs, DEspR+/CD133+ co-expression is detected at the invasive tumor-leading edge, in contrast to minimal, if any, DEspR and CD133 expression in normal pancreatic ducts, parenchyma, and microvessels (Figure 2B). Similarly, glioblastoma tissue showed increased DEspR-positive expression in tumor cells and microvascular endothelium, and co-localized with CD133, a marker for GBM-CSCs [31], thus suggesting DEspR+/CD133+ putative CSCs close to microvessels (Figure 2C), consistent with the microvascular-CSC niche paradigm [17], [18]. In contrast, minimal to no expression was observed in adjacent normal brain tissue, and minimal to no background in isotype-AF568 immunostaining (Figure 2C). These observations are confirmed on analysis of more tumor biopsy cores on a tumor tissue array for pancreatic cancer and glioblastoma detecting a gradient of increased DEspR+ expression in tumor cells, tumor microvessels and invasive tumor cells, although with some GBM-biopsy cores negative for DEspR-expression similar to adjacent normal tissues (Figure S2B).

### In vitro analysis of DEspR roles in angiogenesis, invasiveness and CSC-anoikis resistance

Having validated the 7c5b2 mAb and the experimental cell system for DEspR expression and signal transduction, we studied DEspR roles as a common receptor coordinating mechanism for tumor vessels, nonCSC-tumor cells, and CSCs by analyzing the effects of anti-DEspR 7c5b2 mAb-inhibition in established surrogate in vitro assays for pro-metastatic paradigms: angiogenesis in HUVECs, invasiveness in pancreatic cancer Panc1-cells, and CSC-anoikis resistance, stemness, self-renewal, survival, and tumorigenicity in functionally validated anchorage-independent CSCs derived from Panc1 and glioblastoma-U87 cell lines. These anchorage-independent Panc1 and U87 CSCs represent multiple CSC-subsets in order to better model human tumor heterogeneity [3] in contrast to marker-specific cell-sorting for CSC-subset-specific isolation [32]. Notably, CSCs from both Panc1 and U87 cancer cell lines have been characterized in studies of tumor progression and metastasis events [32]–[34].

**Figure 3:**
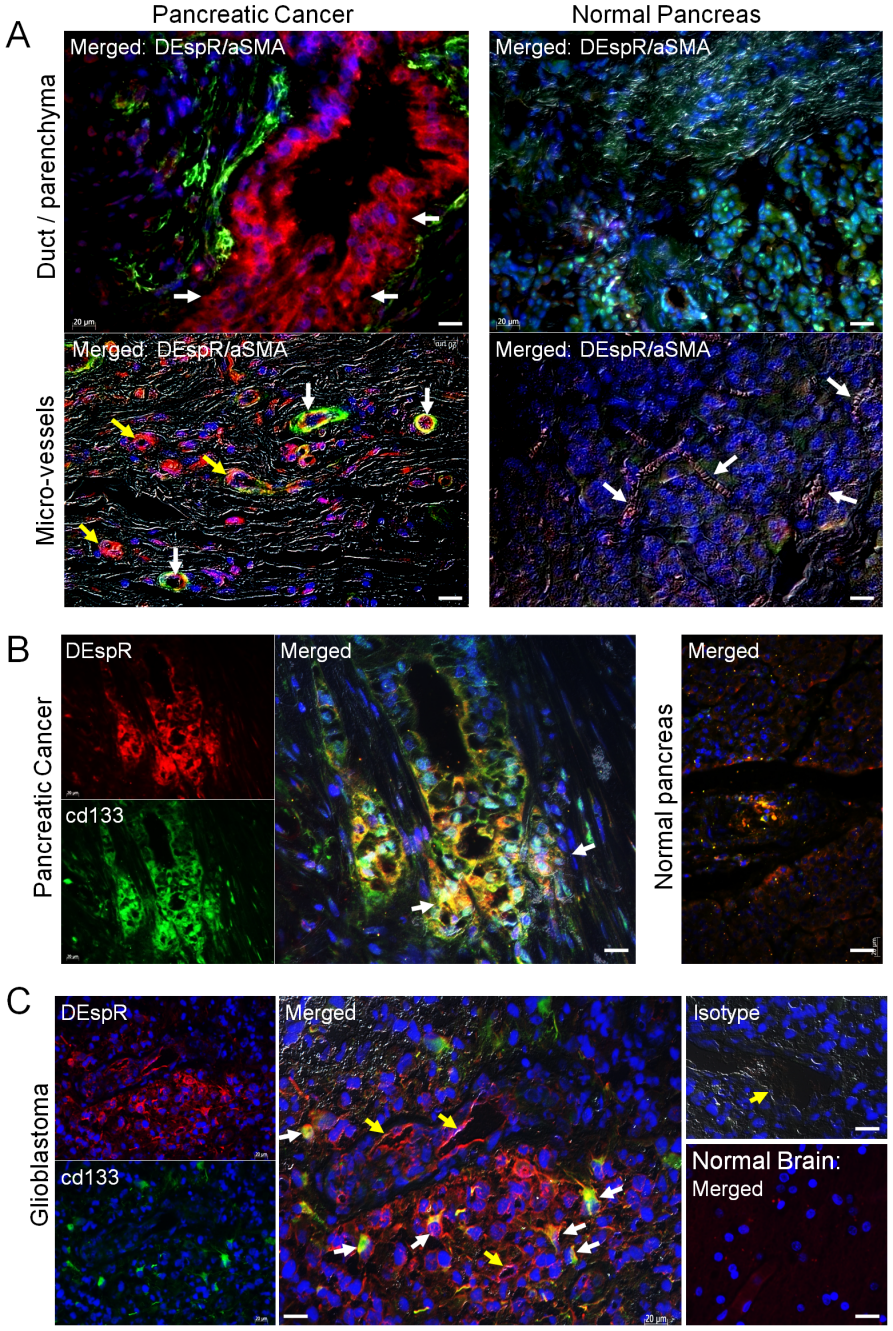
In vitro analysis of DEspR roles. (A) DEspR-positive (red) immunostaining of HUVECs undergoing angiogenic ‘tube’ formation (white arrows) in contrast to DEspR-negative quiescent HUVECs (DAPI-stained blue nuclei). DEspR+ expression in HUVECs forming angiogenic networks. (B) Representative HUVECs angiogenic network formation in Matrigel in control mock-treated angiogenic conditions (Mock Tx) in contrast to decreased angiogenic networks upon DEspR-inhibition (Anti-DEspR Tx). (C) Dose response curve of anti-hDEspR(7c5b2) mAb inhibition of angiogenesis measured as tube length (black circle) and number of branch points (open circle). DEspR-inhibition at 500 nM dose of total angiogenic tube length (Tube length) and mean number of branch points (# Branches) comparing control untreated (C), pre-immune negative control (PI) for polyclonal anti-hDEspR ab (Pab), isotype negative control (Iso) and for anti-hDEspR 7c5b2 (Mab). Anti-angiogenic effects in rat aortic ring assays (Figure S3). (D) Representative immunofluorescence analysis of nonCSC adherent Panc1-cells; DEspR-positive (green) immunostaining (DEspR), CSC-marker CD133-positive immunostaining (red). Merged DEspR and CD133 immunostaining with differential interference contrast (DIC) overlay showing co-expression in subset of Panc1-cells (yellow-green) forming clusters mounding above the culture-dish plane; DAPI nuclear stain (blue). (E) Anti-hDEspR 7c5b2 inhibition of Panc1 cell invasiveness; C, non-treated cells, Iso, isotype IgG2b mock-treated cells, Mab, anti-hDEspR 7c5b2 mAb at 500 nM. *, P<0.0001 one-way ANOVA with Tukey multiple comparisons testing of anti-DEspR antibodies with respective controls; bar, 20 microns.

To define DEspR functional roles in tumor angiogenesis, we demonstrate that DEspR is increased in tube-forming angiogenic HUVECs but not in quiescent HUVECs (Figure 3A), and that anti-DEspR (7c5b2)-inhibition decreased angiogenic tube length and branch formation (Figure 3C) in a dose-response manner (Figure 3C) in HUVECs grown in angiogenic culture conditions. To confirm these findings, parallel studies in rat aortic ring angiogenesis assay were performed showing that DEspR-inhibition via a neutralizing anti-ratDEspR polyclonal antibody inhibited both ET1- and VEGFsp-stimulated angiogenesis (Figure S3).

To first define DEspR roles in invasiveness using adherent nonCSC-Panc1 tumor cells, we demonstrate DEspR-expression in Panc1 cells used in the transwell invasiveness assays since CSCs cultured in suspension preclude the use of established transwell invasiveness assays. Consistent with confocal microscopy analysis (Figure 1D), double immunostaining of nonCSC Panc1 cells detected co-expression of DEspR and CD133, a reported marker of PDAC-CSCs [35], suggesting DEspR-expression in Panc1 non-CSCs and enriched in CD133+ Panc1 subset of putative CSCs. Using an established transwell assay for invasiveness through matrigel, DEspR-inhibition via anti-hDEspR (7c5b2) treatment decreased invasiveness significantly compared to control non-treated and isotype mock-treated controls (Figure 3E).

**Figure 4:**
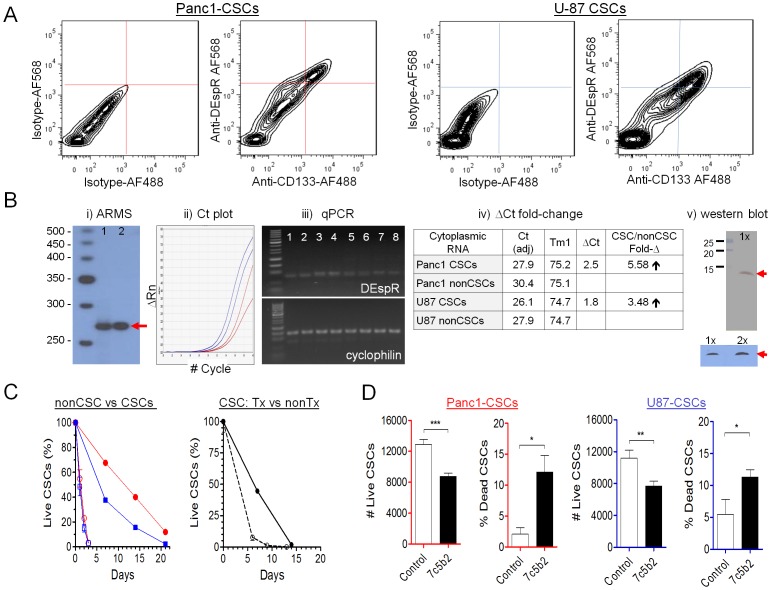
DEspR expression and roles in Panc1-CSCs and U87-CSCs. (A) FACS analysis of anchorage-independent Panc1-CSCs and U87-CSCs comparing control double isotype immunophenotyping (IgG2b-AF568 for 7c5b2 and IgG1-AF488 for CD133) with double anti-DEspR-AF568 and anti-CD133-AF488 immunophenotyping. (B) B-i. ARMS assay specific for spliced DEspR-RNA 270-bp amplicon (red arrow) spanning the spliced exon-to-exon junction (Figure S4B,C) confirms presence of spliced DEspR-RNA in Panc1 (lane 1) and U87 (lane 2) CSCs. B-ii. Quantitative RT-PCR Ct plot. B-iii. Corresponding agarose-gel size fractionation of RT-PCR products demonstrating expected-size DEspR-specific 88-bp amplicon, and control cyclophilin-specific 145-bp amplicon band. DNA size standards: 50-bp DNA ladder). B-iv. Calculation of qRT-PCR ΔCt fold-change in both Panc1- and U87-anchorage independent CSCs. B-v. Western blot analysis detects predicted ∼10 kDa DEspR protein (red arrow) in membrane proteins isolated from U87 CSCs in three independent experimental conditions: 1X, 1∶100 anti-DEspR ab-dilution in two different detection systems; 2X, 1∶50 dilution of anti-DEspR mAb in ECL detection system. (C) Analysis of survival in adverse conditions comparing Panc1- (red open circle) and U87- (blue open square) nonCSCs with quinary CSCs: Panc1 (red circle) and U87 (blue square). Analysis of effects of anti-DEspR (7c5b2) treatment on U87-CSC survival measured as %-live CSCs comparing quinary 7c5b2-treated U87-CSCs (black open circle) and control non-treated U87-CSCs (black circle) in identical adverse conditions. (D) Analysis of effects of DEspR-inhibition on Panc1- and U87-CSC survival in suspension culture at physiological conditions measured as number of live cells and % dead cells comparing quinary anchorage-independent 7c5b2-treated CSCs (black bars) and control non-treated CSCs (white bars). ANOVA P<0.0001, Tukey's multiple pairwise comparisons tests: ***, P<0.0001; **, P<0.001; *, P<0.05.

Using FACs analysis of freshly isolated, non-permeabilized anchorage-independent CSCs to ascertain DEspR-expression on the cell membrane of Panc1-CSCs and U87-CSCs respectively (Figure 4A), we detected co-expression of DEspR and CD133, a known PDAC and GBM CSC-marker [35]. CD133+ cells were detected in 0.1% to 3% of nonCSC Panc1- and U87-cells respectively (data not shown), in contrast to ≥30% CD133+ CSCs in both Panc1- and U87-CSCs using identical labeling conditions (Figure 4A). Notably, ≥90% of CD133+ CSCs are DEspR+ in both Panc1 and U87-CSCs (Figure 4A).

To confirm DEspR expression in Panc1-CSCs and U87-CSCs, we first corroborated detection of spliced DEspR-specific RNA, to distinguish it from FBXW7 in cancer cells via Amplification Refractory Mutation System (ARMS) assay [36]–[38]. As shown in Figure 4B-i (and S4) ARMS assay specific for spliced –DespR transcript detected the predicted 270 bp amplicon. Figure 4B-i also shows that the ARMS forward primer specific for spliced DEspR-RNA does not produce the 342 bp amplicon representing the unspliced DEspR transcript containing the 72 bp intron (Figure S4). This indicates the specificity of ARMS-test design for spliced DEspR-transcript as expected given that ARMS can detect single nucleotide substitutions [36]–[38]. To confirm increased DEspR expression as observed by FACS analysis (Figure 4A), we performed quantitative real-time reverse transcriptase PCR analysis of cytoplasmic RNA derived from freshly isolated Panc1- and U87 nonCSCs and CSCs. We quantified DEspR RNA using an 88 bp-amplicon primer pair selected for robust quantitative RT-PCR standards (DS Gene 1.5 software). Concordantly, 5.5-fold and 3.5-fold increases in DEspR-specific 88-bp amplicon in Panc1-CSCs and U87-CSCs are detected by qRT-PCR respectively (Figure 4B-ii to iv) which were all confirmed to be DEspR by nucleotide sequencing (data not shown). Western blot analysis was then done to confirm endogenous DEspR protein in CSCs. As shown (Figure 4B-v) western blot detected the predicted size ∼10 kDa, as previously shown [31].

To test for increased CSC survival abilities of Panc1- and U87-derived CSCs, a known feature of CSCs [4], and to test DEspR roles in CSC survival, we analyzed survival in adverse experimental conditions (hypoxia, decreasing pH, and cold-induced metabolic stress) to approximate adverse tumor microenvironment stresses. We compared Panc1 and U87 quinary (P5, passage 5) CSCs and nonCSC tumor cells respectively. As shown in Figure 4C, more CSCs were alive by day 4, in contrast to no live nonCSC tumor cells in identical adverse conditions. Moreover, as shown in Figure 4C right-panel, anti-hDEspR (7c5b2) mAb treatment of CSCs in identical adverse conditions decreased CSC survival 5-fold, with only 10% of treated CSC tumor cells alive by day 6, in contrast to more than 50% live CSCs in control non-treated CSCs. Together, these data demonstrate a requirement of DEspR functionality for increased-survival of CSCs in these representative experimental adverse conditions.

To test DEspR roles in anoikis resistance, a requirement for metastasis and local invasiveness pertinent to all cancers especially pancreatic cancer and glioblastoma [39]–[41], we analyzed inhibition of anoikis resistance by anti-hDEspR (7c5b2) treatment of CSCs in standard anchorage-independent spheroid-culture conditions. Compared to non-treated control CSCs, DEspR-inhibition decreased CSC survival in suspension culture conditions, an established in vitro test for anoikis resistance, in treated Panc1-CSCs and treated U87-CSCs, measured as increased number of dead CSCs and decreased number of live CSCs (Figure 4D). Concordantly, 16-hour DEspR inhibition of Panc1- and U87-CSCs in anchorage-independent culture conditions increased the balance towards pro-apoptotic gene expression by net increased expression of pro-apoptotic genes and decreased anti-apoptotic genes (Figure S4A). Together these results suggest a role for DEspR in anoikis resistance required for tumor local invasion and distant metastasis.

### In vivo DEspR-inhibition effects on Panc1-CSC and U87-CSC xenograft tumors

Having shown DEspR-inhibition effects on individual cancer paradigms, we next tested the “common-receptor coordinating paradigm” by determining the in vivo integrated effects of coordinated inhibition of angiogenesis, tumor cell invasiveness, and CSC anoikis resistance or survival via anti-DEspR mAb therapy. In order to test larger tumor sizes than that attainable in orthotopic nude mouse or nude rat tumors, we analyzed heterotopic PDAC Panc1-CSC and glioblastoma U87-CSC subcutaneous tumors in nude rats.

**Figure 5:**
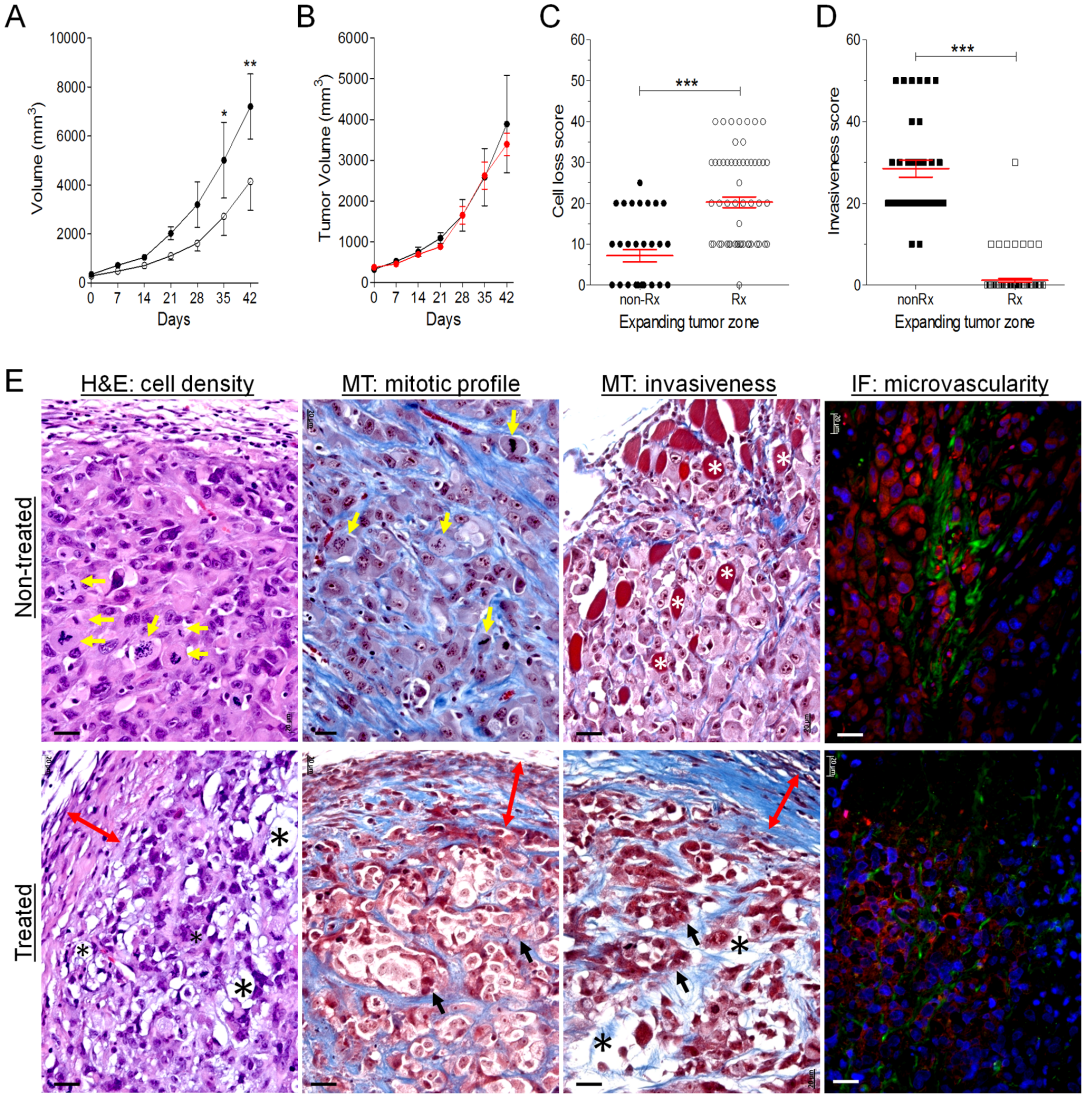
Anti-tumor effects of DEspR-inhibition in Panc1-CSC xenograft tumors in nude rats. (A) Comparative analysis of tumor volumes (mm^3^) of anti-DEspR mAb treated Panc1 CSC-xenograft tumors (open circle, n = 6) in contrast to concurrent non-treated controls (black circle, n = 3). Repeated measures ANOVA with Tukey's multiple comparison test: *, P<0.05; **, P<0.01. (B) Comparative analysis of gemcitabine treatment (red circle, n = 5) and concurrent non-treated (black circle, n = 4) controls. (C) DEspR-inhibition of Panc1-CSC xenograft tumors increased cell loss-scores in the expanding tumor zones and (D) reduced invasiveness-scores (one-way ANOVA with pairwise Tukey's multiple comparison test, ***, P<0.0001). (E) Representative images of tumor expanding zones with identical exposure settings depict robust differences in key histopathological parameters pertinent to efficacy determinations in Panels A, C and D between non-treated control (Non-treated) and anti-DEspR mAb treated (Treated) nude rat subcutaneous Panc1-CSC xenograft tumors: 1) cellularity (H&E: Cell Density, 2) mitotic index (Masson Trichrome MT: mitotic profile), 3) extent of tumor invasiveness to adjacent normal tissue (MT: invasiveness), and 4) human-specific CD31+ and human-specific 7c5b2 DEspR+ fluorescent immunostaining of microvessels and adjacent tumor cells (IF: microvascularity). (yellow arrows, mitotic cells; white asterisk, skeletal muscle cells amidst invading tumor cells; red double headed arrows, fibrotic tumor capsule; black arrows, tumor islands; black asterisk, cell loss with decrease collagen density. Immunofluorescence (IF) DEspR (red), CD31 (green); DAPI nuclear stain (blue).

**Figure 6:**
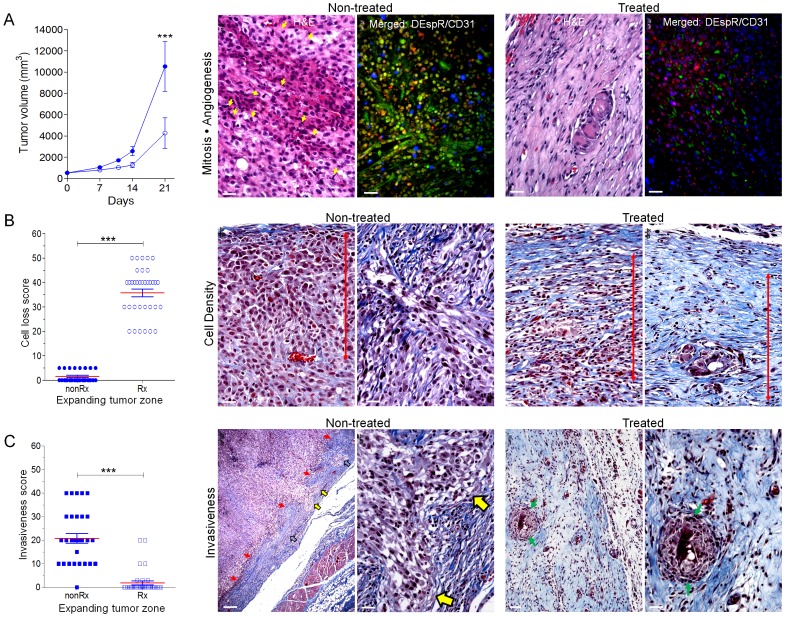
Anti-tumor effects of DEspR inhibition in U87-CSC xenograft tumors. (A) Line plot of subcutaneous U87-CSC xenograft tumor volumes comparing treated (n = 6) and non-treated control (n = 4) nude rats (repeated measures ANOVA, Tukey's multiple comparisons test, P<0.001). Representative histopathological images of Non-treated and Treated xenograft tumors depict differences in Mitosis-Angiogenesis in the tumor expanding zone, H&E, greater numbers of mitotic cells (yellow arrows), glomeruloid vessels, and numerous CD31+ microvessels and cells (Merged DEspR/CD31 immunofluorescence). DEspR+, red; CD31+, green, DEspR+/CD31+ co-expression yellow/orange; bar, 20-microns. (B) DEspR-inhibition increased cell loss-scores (P<0.0001, ANOVA with Tukey's MCT) in treated rats compared to non-treated controls. Representative histopathological Masson-trichrome (MT) images show greater tumor cell density and palisading tumor cells in non-treated U87-CSC xenograft tumors compared to treated tumors with decreased cell density and greater collagen deposition around tumor cell ‘islands’ (yellow arrows in C). Red double headed arrow depicts equidistant area from tumor capsule; bar, 20-microns. (C) DEspR-inhibition decreased Invasiveness scores (P<0.0001, ANOVA with Tukey's MCT) in the expanding tumor zone in treated rats compared to non-treated rats. Representative histopathological MT-stained images with greater invasiveness through the tumor capsule (red arrows, yellow arrows) in non-treated tumors compared to treated rat tumors [tumor ‘islands’ surrounded by amorphous substances (light blue) with some collagen (darker blue)]. Paired red-black arrows, tumor capsule invaded by U87 cells; green arrows, tumor islands. Bar, 50-microns left panels; 20-microns right panels respectively for non-treated and treated tumor sections.

As shown in Figures 5–6, and Figures S5–S6, the prerequisite tumor model validation is attained via histopathological and immunofluorescence analysis for both Panc1-CSC and U87-CSC xenograft tumor models. Briefly, Panc1-CSC xenograft tumors exhibited geographic necrosis (not shown) and an expanding tumor zone comprised of high-grade, poorly differentiated tumor cells with high mitotic index, pericellular collagen deposition around some tumor cells that are reminiscent of stromal desmoplasia, high human-specific DEspR expression in some but not all tumor cells consistent with tumor heterogeneity, and human-specific DEspR expression in intratumoral blood vessels indicating Panc1-CSC tumor vasculogenesis similar to glioblastoma-stem cell vascular-transdifferentiation [9]–[12]. Notably, rat-specific anti-DEspR mAb did not immunostain tumor cells and intratumoral microvessels (Figure S5D). Similarly, U87-CSCs exhibited robust tumorigenicity with almost 100% subcutaneous tumor production in nude rats. These heterotopic xenograft tumors exhibited typical areas of necrosis (data not shown) with or without pseudo-palisades of tumor cells, vascularized expanding tumor zone comprised of high-grade, poorly differentiated tumors cells with high mitotic index, and microvascular proliferation with glomeruloid structures (Figure 6A, Figure S6B, S6D). Notably, U87-CSC tumors exhibited invasion through the fibrous cap (Figure 6C) and into surrounding skeletal muscle tissue (Figure S6A), in contrast to reported non-invasiveness of nonCSC-U87 mouse orthotopic xenograft tumors [42]. Similar to Panc1-CSC xenograft tumor observations, we detected DEspR+ immunostaining of U87-CSC xenograft tumor cells in the expanding zone and invasive edge, as well as DEspR+ intratumoral vessels (Figure 6A, S6B) thus placing DEspR at the CSC-microvascular niche [15], [18]. These observations also indicate CSC-derived tumor vasculogenesis given 7c5b2 mAb-specificity for human-DEspR [31]. Detection of DEspR-positive and DEspR-negative tumor cells indicate tumor cell heterogeneity. Specificity of immunostaining is demonstrated by negative isotype immunostaining (Figure S6C).

After validating Panc1-CSC and U87-CSC xenograft heterotopic tumor modeling, we next tested the in vivo effects of DEspR-inhibition. As shown in Figure 5A, anti-DEspR treatment (both anti-rat and anti-human DEspR inhibition) decreased Panc1-CSC xenograft nude-rat tumor growth compared to non-treated controls, P<0.01, in contrast to gemcitabine failure using standard human doses of gemcitabine (Figure 5B). Additionally, analysis of cell loss and invasiveness in multiple high power fields in the expanding tumor zone revealed significant differences in cell loss (Figure 5C) and invasiveness (Figure 5D), between treated and untreated rats, ANOVA with Tukey's multiple comparisons test, P<0.0001 for both. Histological H&E analysis of treated tumor reveals hypochromatic tumor cells, indicative of dying tumor cells in the expanding tumor zone with a thicker, collagen-rich fibrous cap (Figure 5E, Treated) in contrast to control non-treated tumors with high mitotic index and robust tumor cell proliferation in the expanding zone with thin or absent fibrous tumor caps (Figure 5E, Non-treated). Masson-trichrome stained sections confirm the thicker fibroblast-rich fibrous caps, increased tumor cell loss, decreased invasiveness in the expanding tumor zone of treated rat tumors in contrast to non-treated controls with invasiveness extending through the cap, and in some tumors through to the adjacent muscle tissues (Figure 5E). To ascertain the human-CSC origin of xenograft tumor microvasculature, we performed immunofluorescence analysis using human-specific anti-CD31 mAb. We detected CD31+ microvessels among DEspR+ tumor cells, which were noticeably decreased in anti-DEspR treated Panc1-CSC xenograft tumors compared with non-treated control tumors (Figure 5E).

Similarly, compared to non-treated controls, anti-DEspR mAb treatment decreased U87-CSC glioblastoma xenograft tumor growth P<0.001 (Figure 6A) and invasiveness P<0.0001 (Figure 6C), as well as increased cell loss, P<0.0001 in the expanding tumor zones (Figure 6B). Representative images demonstrate higher tumor vascularization, cell density and greater invasiveness in non-treated tumors (Figure 6, Non-treated panels) compared to treated xenograft tumors with less vascularization, decreased tumor cell density, increased fibrosis surrounding residual “tumor cell islands” in the expanding tumor zone (Figure 6, Treated Panels). Furthermore, there is decreased CD31+ tumor microvessels in treated xenograft tumors and decreased DEspR+ expression in tumor cells compared to non-treated xenograft tumors (Figure 6A). Interestingly, significant number of U87-CSC xenograft tumor cells co-express DEspR and CD31 especially in areas surrounding CD31+ microvessels (Figure 6A), similar to observations in patient-derived glioblastoma-stem cell xenograft tumor models [12]. Furthermore, while glomeruloid microvessels are thin walled in non-treated tumors (Figure S6D), microvessels in treated tumors exhibit contiguous endothelial walls (Figure S6E) suggesting stabilization of leaky angiogenic blood vessels.

## Discussion

Here we show that DEspR is a common receptor expressed in tumor cells, microvessels, and anchorage-independent CSCs, with differential expression in cell- and nuclear-membranes, as well as in the cytoplasm. DEspR is differentially increased in both human pancreatic cancer and glioblastoma in contrast to adjacent normal tissue. This increase is detected at the protein and RNA levels in Panc1- and U87-CSCs.

Because of the overlapping transcript organization, DEspR inhibition studies presented here are all done at the protein level, in order to eliminate potential dual DEspR/Fbxw7 knockdown-inhibition at the RNA level. DEspR-inhibition at the protein level decreased in vitro angiogenesis, tumor cell invasiveness, CSC-cell anoikis resistance, survival, and promoted pro-apoptosis balance for both Panc1-CSCs and U87-CSC experimental systems. Concordantly, DEspR-inhibition also decreased in vivo Panc1- and U87-CSC-xenograft tumor volumes, vasculogenesis, invasiveness, and tumor cell survival in the expanding tumor zone. In contrast, the current standard of care for pancreatic cancer, gemcitabine, given intravenously at human equivalent dose had no effect on tumor growth of Panc1-CSC xenograft tumors, thus indicating the potential of DEspR-targeted inhibition for improved clinical therapy for gemcitabine-resistant pancreatic cancer. On the other hand, a standard of care chemotherapy for GBM, temozolomide, prevented early tumor growth of U87 CSC-xenograft tumors (data not shown), but comparative analysis of temozolomide and DEspR-inhibition in tumor progression associated with larger tumors, and in temozolomide-resistant U87-CSCs remain to be studied. Nevertheless, DEspR-inhibition resulting in decreased invasiveness observed in both Panc1-CSC and U87-CSC derived xenograft tumors is of translational importance since both gemcitabine and temozolomide are not effective in invasive PDAC and GBM, respectively. Further testing of DEspR-inhibition on tumor cell invasiveness and tumor vasculo-angiogenesis in orthotopic xenograft tumor models for both PDAC and GBM will be important.

The concurrent decrease in invasiveness, tumor vasculo-angiogenesis, and survival in the expanding tumor zones in both Panc1-CSC and U87-CSC xenograft tumors collectively support DEspR as a common receptor mechanism whose activation on different cancer cell players would facilitate contemporaneous tumor progression. Given the complexity of cancers and the expected redundancy of multiple survival, vasculogenic and invasiveness pathways, these data indicate nodal functional significance of DEspR roles in said tumor progression events in both PDAC and GBM.

These observations and deductions are strengthened by the fact that septenary tumorigenic CSCs used for study at 2×10^6^ per xenograft injection exhibited the expected increased tumorigenicity over non-CSC cancer cell lines, xenograft tumor cell heterogeneity and invasiveness, and reached tumor sizes greater than 2000 mm^3^ 42 days after cell-inoculation. In contrast, in nude mice at ∼1/10 the size of nude rats, injection of 5×10^6^ Panc1 cells were used for tumor formation which reached an average 1700 mm^3^ only, 71 days post-injection [43]. Interestingly, CSC-xenograft tumors exhibited tumor vasculogenesis for both U87- and Panc1-CSCs, while differentially recapitulating the typical high microvascular density of glioblastoma in contrast to microvascular paucity and stromal collagen deposition of pancreatic cancer in humans. These data suggest that both Panc1-CSCs and glioblastoma U87-CSCs isolated through anchorage-independent cultures and comprised of mixed CSC immunophenotypes, can drive tumor vasculogenesis and maintain tissue-type-specific characteristics in xenograft tumors despite heterotopic microenvironments.

Furthermore, these observations are concordant with DEspR-mediated signaling. The combinatorial activation of Src and FAK-phosphoproteins is associated with invasiveness and metastasis [44], [45], while the dual Src- and STAT3-activation is associated with resistance to chemotherapy [46]. Moreover, DEspR-activation of Akt [30], Src, STAT3 and BRCA1 (Figure 1B) comprise phosphoproteins independently shown by others to induce HIF-1alpha [47]–[50], predicts an autocrine feed forward loop for constitutive activation of HIF1a and DEspR once HIF-1alpha is activated by hypoxia, given that HIF1a is known to increase ET1 and VEGF [51], [52], and hence VEGFsp. This predicted positive feedback loop between DEspR-activated Akt, Src, FAK, STAT 3 and HIF-1alpha activation suggests the hypothesis that DEspR activation can contribute to the observed constitutive activation of these established pro-malignancy signaling networks [44], [45], [53]–[58] even after tumor hypoxia is decreased or resolved.

Altogether, in vitro and in vivo functional analysis of DEspR supports a “common receptor paradigm” for contemporaneous CSC survival, anoikis resistance, invasiveness and tumor vasculogenesis at the CSC-microvascular niche and invasive tumor edge. We hypothesize that this could comprise a putative trans-cellular mechanism for temporal coordination beyond stochasticity. As an accessible cell-surface, coordinating receptor on nonCSC tumor cells, CSCs and tumor microvessels involved in key tumor-progression events, DEspR inhibition opens a novel targeted therapy approach for pancreatic cancer and glioblastoma.

## Materials and Methods

### Ethics statement

This study was performed in strict accordance with the recommendations in the Guide for the Care and Use of Laboratory Animals of the National Institutes of Health. The protocol was approved by the Committee on the Ethics of Animal Experiments of Boston University School of Medicine (Permit Number: AN-15160). Euthanasia of study animals was done by removal of vital organs and exsanguination under general anesthesia as stated in AVMA Guidelines 2013.

### Cell lines and antibody development and characterization by ELISA

U87 glioblastoma and Panc1 cells were obtained from American Type Culture Collection, ATCC (Manassas, VA) and maintained according to ATCC-specifications. Human umbilical vein endothelial cells, HUVECs, were obtained from Cascade Biologics, Inc. and maintained according to CBI-specifications. Monoclonal antibody development was custom performed by ProMab Biotechnologies, using a humanDEspR-specific nine amino-acid peptide, M_1_TMFKGSNE_9_ at the amino-terminal end of hDEspR as antigen. This antigenic-peptide was previously validated via the development of polyclonal anti-hDEspR ab which detected the expected size product of transcribed, spliced, and translated hDEspR expression construct in permanent Cos1cell transfectants [31]. Screening of hybridoma supernatants and characterization of monoclonal antibodies were performed by ELISA using M_1_TMFKGSNE_9_ antigenic peptide. Serial dilutions of primary antibodies were incubated at 37°C for 1 hr. The wells were then incubated with horse radish peroxidase (HRP)-labeled anti-IgG (Sigma) at 37°C for 1 hr. Reactions were analyzed at 450 nm after addition of 3,3′5,5′-tetramethylbenzidine substrate at 37°C for 10 minutes.

### Multiplex analysis of signaling proteins by Ab-microarray

Analysis of ligand-dependent modulation of different signaling pathways by DEspR was custom performed (Kinexus) utilizing the Kinex™ Antibody Microarray System: 506 phosphoprotein-specific antibodies in duplicates or multiple replicates, and 740 pan-specific antibodies of signaling molecules. Effects of ET1- and VEGFsp-DEspR activation on multiplex signaling pathways after 30 minutes of ligand-treatment (ET1, 10 nM; VEGFsp, 10 nM) was compared with non-activated, non-treated controls, using Cos1-hDEspR permanent cell transfectants developed previously [31]. Data are presented as %-change from control (% CFC) after 30-minutes of ET1 or VEGFsp-treatment compared with non-treated transfectant-matched controls respectively. The %-CFC = [Treated^Ave^−Control^Ave^]/Control^Ave^×100.

### Immunofluorescence analyses of cells and tumor tissue sections

Double immunofluorescence staining was done as described [59]. Rat-specific anti-DEspR mAb (10a3h10) and human-specific anti-DEspR mAb (7c5b2) were labeled with AlexaFluor(AF)-488 or AF568, and used at 10 ug/ml for cells or frozen sections, and at 100 ug/ml for fixed, paraffin-embedded sections following antigen-retrieval. Anti-alpha-smooth muscle actin, aSMA (SIGMA-ALDRICH, MO), anti-CD133 (Creative Biomart, NY) and human-specific anti-CD31 (Sta. Cruz Biotechnology, TX) were used per manufacturer's specifications. For quantitation of immunofluorescence results of tumor biopsy cores, digital photomicroscopy was performed using a Zeiss Axioskop fluorescence microscope using identical exposure settings for tumor sections and normal controls once ideal settings determined for positive target-specific fluorescence. For quantitation of relative fluorescence intensity levels, auto-exposure times in milliseconds were recorded using identical microscopy settings in photomicroscopy sessions.

### Isolation of cancer stem-like cells (CSCs) from Panc1 and U87 cell lines (ATCC)

Panc1 and U87-cells in log phase were harvested and subcultured in complete MammoCult® medium (Stem Cell Technologies, BC, CANADA) in 5% CO2 humidified incubator at 37°C. After 2–3 weeks in culture, Panc1 and U87 cells were harvested and plated in complete MammoCult® medium containing 0.5% Methylcellulose (Stem Cell Technologies, BC, CANADA) in 100 mm ultra-low attachment plates. In vitro experiments were performed using quinary CSCs; xenograft tumors were developed from septenary CSCs. Functional validation of CSC stocks was done through demonstration of maintenance of self-renewal in suspension cultures and anchorage independence through 7 passages, increased tumorigenicity over non-CSC cells, tumor vasculogenesis and tumor cell heterogeneity in xenograft tumors.

### FACS analysis of Panc1-CSCs and U87-CSCs

Panc1-CSCs and U87-CSCs were incubated in ice-cold Hank's balanced salt solution (HBSS, Invitrogen, NY) plus 2% FBS containing: a) 10 µg/ml AF-568 labeled 7c5b2 mAb plus 10 µg/ml AF-488 labeled anti-CD133 (Creative BioMart, NY); b) 10 µg/ml AF-568 labeled IgG2b (isotype) plus 10 µg/ml AF-488 labeled IgG1 (isotype). Duplicate samples were incubated for 20 min at 4°C, washed, resuspended in 1% FBS/HBSS, 1% PFA, filtered and analyzed on an LSR-II-FACS instrument. Analysis was done using FloJo Flow Cytometry Analysis Software (www.FloJo.com).

### FACS analysis of competition ligand-7c5b2 mAb binding to Panc1-CSCs

Panc1-CSCs were incubated in ice-cold Hank's balanced salt solution (HBSS, Invitrogen, NY) containing 2% FBS, 10 µg/ml AF-568 labeled 7c5b2 mAb in the absence of competitors or in the presence of 50-fold molar excess of either ET1 or VEGFsp respectively. Duplicate samples were incubated for 20-minutes at 4°C, processed and analyzed on an LSR-II FACS instrument.

### Saturation binding of 7c5b2 mAb to DEspR on Panc1 CSCs

7c5b2 mAb was labeled with Alexa Flour 568 using the Alexa Fluor 568 Monoclonal Antibody Labeling Kit (Invitrogen). Specific binding was determined by FACS analysis using 10^5^ cells in 0.25 ml of Hank's balanced salt solution containing 2% FBS and increasing concentrations of 7c5b2 mAb (1–100 µg/ml). Incubations were done at 4°C for 20 min and immediately subjected to FACS analysis on a BD™ LSRII Flow cytometer. Each data point was performed in duplicate.

### HUVEC tube formation assay for angiogenesis

Angiogenesis assays were done using human umbilical vein cells, HUVECs (Cascade Biologics, NY) using standard angiogenesis conditions with 20,000 cells per well in a 96 -plate Angiogenesis System: Endothelial Cell Tube Formation Matrigel™ Matrix (BD Biosciences, CA). Different anti-angiogenic conditions were assayed in quadruplicate: basal media (BM) alone (C, control); increasing concentrations of 7c5b2 mAb (0.05–500 nM); BM with 500 nM IgG2b isotype control; BM with anti-hDEspR polyclonal antibody (1∶200); BM with pre-immune serum (PI, 1:200). After 16 hours, resulting tube formations were analyzed using ImageJ (NIH).

### Invasion assay

Panc1-cell invasion assays were performed as described [60] using the BD Bio-Coat Matrigel invasion assay system (BD Biosciences, CA). Panc1-cells were suspended in serum-free DMEM and seeded onto pre-coated transwell chambers. The transwell chambers were then placed into 24-well plates, to which basal medium only (C, control) or basal medium containing IgG2b isotype control (500 nM) or basal media containing anti-hDEspR(7c5b2) mAb (500 nM) were added. DMEM containing 10% FBS was used as chemoattractant. After 16 hours, the number of stained-invading cells per well were counted under the microscope. Each condition was assessed in four replicates.

### Quantitative real-time PCR (qRT-PCR)

Panc1 cells, U87 cells, Panc1 CSCs and U87 CSCs were lysed with ice-cold Nuclei EZ lysis buffer (SIGMA-ALDRICH, MO) and supernants containing cytoplasmic RNA were extracted twice with Phenol:Chloroform (50∶50) followed by ethanol precipitation. Total cytoplasmic RNA concentration was determined by absorbance at 260 nm. After removal and documentation of no residual DNA contamination, cDNA synthesis was performed with the QuantiTect Reverse Transcription Kit (Qiagen, MD) using 400 ng of total cytoplasmic RNA, a DEspR specific primer (Reverse R1^88 bp^: 5′-TGGACCAGAGAAATTGCTTG-3′, Figure S1A) and a Cyclophilin specific primer (Reverse: 5′-GAAGTCACCACCCTGACA-3′). Amplification was performed with the QantiTect SYBR Green PCR Kit (Qiagen, MD) using the StepOnePlus PCR System (Applied Biosciences, CA) and the following two primer sets: for DEspR (Forward F1: 5′-GGGGTTCTATCACTTGCATC-3′, Figure S1A; Reverse R1^88 bp^: 5′-TGGACCAGAGAAATTGCTTG-3′, 88 bp amplicon, Figure S1A) and for endogenous control, Cyclophilin A (Forward: 5′-GCGTCTCCTTTGAGCTGTT-3′; Reverse: 5′-GAAGTCACCACCCTGACA-3′, 145 bp amplicon). qRT-PCR and cycling parameters were as follow: cDNA synthesis for 15 min at 42°C, thermal inactivation of reverse transcriptase for 3 min at 95°C and 40 cycles of PCR (melting for 15 sec at 94°C, annealing 30 sec at 55°C, synthesis for 30 sec at 72°C). Data acquisition and analysis were carried out using StepOne software v2.0 (Applied Biosciences, CA).

### Western blot analysis

Western blot analysis was done essentially as described [31] using 30 µg of cell membrane proteins isolated from U87 CSCs and a mouse mAb (5G12E8; 80 µg/ml, 14 hours at 4°C) raised against the identical human DEspR-specific peptide M_1_TMFKGSNE_9_ used to develop 7c5b2 mAb. We note that 7c5b2 does not perform well in Western blots, but 5G12E8 does. Immunoreactive proteins were detected by DAB using the Metal Enhanced DAB Substrate kit (Thermo Scientific, MA) or by chemiluminescence using the ECL Western Detection kit (Thermo Scientific, MA).

### Detection of spliced DEspR-specific mRNA by using the Amplification Refractory Mutation System (ARMS) assay

ARMS was performed as described [36]–[38] using DEspR-specific single strand cDNA synthesized from total RNA isolated from Panc1- and U87- CSCs. cDNA synthesis was done with 5 µg of total RNA using the DEspR-specific reverse primer 5′-AGGAGCCACTTTTTATACAGTTCTACCCTGATCAAC-3′ and thermo-stable Maxima Reverse Transcriptase as per manufacturer's instructions (Thermo Scientific, MA). Reactions were incubated at 65°C for 2 hours followed by residual RNA hydrolysis at 37°C for 14 hours in 0.5 N NaOH, subsequent neutralization with 1 N HCl, ethanol precipitation, and resuspension in 20 µL of sterile water. We used 2 µL of this DEspR-specific single-strand cDNA stock for PCR-amplification with the forward primer specific for spliced DEspR: 5′-CATGACAATGTTTAAAGG**GAGC**-3′; and the reverse primer being the identical primer used for DEspR-specific cDNA synthesis. Using this primer pair, ARMS amplicon should be 270 bp specific for spliced DEspR (Figure S4B). Detection of spliced-specific amplified products (270 bp) was done by ^32^P-end labeling the spliced DEspR-specific forward primer and subsequent size fractionation on a 6% denaturing polyacrylamide gel. The optimal stringent PCR cycling conditions experimentally determined were as follows: 95°C for 15 min; 40 cycles of 94°C×15 sec, 57°C x 30 sec, 72°C×30 sec; and extension at 72°C×7 min. Reaction volumes were 10 µL, using 0.7 µmol/L of each primer and the QuantiTect SYBR Green PCR kit (Qiagen, MD).

### Survival of cancer cells and CSCs in adverse conditions

Non-CSC and anchorage-independent CSCs were incubated in respective media in adverse conditions (in test tubes, at 4°C, no CO2 incubator; no media change.) For Panc1 and U87 nonCSC tumor cells, live cells were counted at day-1, 2 and 3 post-set up using Trypan Blue. For CSCs, live CSCs were counted at day-7, 14 and 21 post-set up using Trypan Blue. To test DEspR roles in survival, one million U87-CSCs were incubated in duplicates in the absence (control) or presence of anti-DEspR 7c5b2 mAb (500 nM added once at day 0) and live U87 CSCs counted at various times using Trypan Blue.

### DEspR-inhibition studies of Panc1-CSC and U87-CSC anoikis resistance and survival

CSCs (2000/well) were seeded in ultra-low attachment 96-well plate with 500 nM 7c5b2 (treated) and without (control) with 6 replicates. CSCs were cultured in optimal (5%CO2, humidified incubator at 37°C) non-adherent conditions. Anti-hDEspR (7c5b2) mAb was added at seeding, day-2 and day-4. Live and dead CSCs were counted using Trypan Blue on day-5.

### Pathway-focused gene expression profiling

1×10^5^ Panc1-CSCs and 1×10^5^ U87-CSCs were grown in 2 ml of complete MammoCult® growth medium without methylcellulose in the presence (500 nM) or absence of 7c5b2 mAb for 24 hours. RNAs from the treated and non-treated samples (controls) were extracted with Trizol reagent (Invitrogen) as described [61]. We used the human Apoptosis RT^2^ Profiler™ PCR Array (SABiosciences, MD) querying 84 genes related to apoptosis. We performed RT-PCR analysis as per manufacturer's instruction using 100 ng of RNA without the amplification step with each sample ran in four technical replicates.

### Tumor studies in Panc1 CSC-derived and U87 CSC-derived xenograft models

Heterotopic subcutaneous xenograft tumor models were developed using 2×10^6^ Panc1-CSCs and U87-CSC in 4–5 weeks-old nude*^nu/nu^* female rats (Charles River Laboratories, MA). Anti-DEspR mAb therapy (n = 6 each for Panc1-CSC and U87-CSC) or gemcitabine treatments (n = 5) commenced at tumor volumes of 300–400 mm^3^. To inhibit both human-CSC and host-rat DEspR, we used anti-humanDEspR(7c5b2)-mAb and anti-ratDEspR(10a3h10)-mAb at 125 µg/kg/dose each intravenously 2×/week x 4-weeks, beginning on day-14 for Panc1-CSC and day-7 for U87-CSC tumors. Gemcitabine was administered to Panc1-CSC tumor rats at 26 mg/kg/dose IV x 4-weeks equivalent to human dose [62] at 1000 mg/m^2^. Contemporaneous mock controls (n = 7 total for Panc1-CSC tumors, n = 4 for U87-CSC tumors) were infused with vehicle (saline) respectively. Treatments ended when the controls reached maximum allowable tumor size: 6-week study for Panc1-CSC and 3-week study for U87-CSC xenograft tumor models. Tumor volumes were calculated by using the formula (4/3πr_1_^2^×r_2_) where r_1_ is the larger, and r_2_ the smaller radius as described [63].

### Expanding tumor zone analysis of invasiveness and cell loss scores

Comparative analysis of xenograft tumors from control non-treated rats exhibiting robust tumor growth (n = 3) and from treated rats with robust response (n = 3) was done for invasiveness and cell loss in the expanding tumor zone. Representative Masson Trichrome stained sections were obtained to distinguish the collagen rich fibrous cap from invading tumor cells migrating from the typically cell-rich expanding tumor zone. Contiguous high power fields (HPF) (N = 10–20) were scored for invasiveness through the fibrous cap and into surrounding host dermis or muscle tissue. Invasiveness score: 0, no invasive tumor cells; 10, few invasive cells into fibrous cap; 20, invasive cells in <50% width of fibrous cap in HPF; 30, invasive cells along full-length of fibrous cap in HPF; 40, full thickness traversed by invasive cells or ‘thin fibrous cap’; 50, invading cells into adjacent host tissues. HPFs scored for invasiveness were also scored for cell loss marked by residual ‘cell-ghosts’ or amorphous substance or fibrotic replacement: 0, no cell loss; 10, some cell loss in HPF beneath fibrous cap; 20, >25% cell loss in HPF; 30: >50%; 40, >75% cell loss; 50, 100% cell loss with fibrosis or amorphous substance in HPF.

### Statistical analysis

All data were analyzed for normality and descriptive statistics. The following statistical tests were performed using SigmaPlot 11.0 or PRISM 5: one-way analysis of variance (ANOVA) followed by Tukey multiple comparisons test (MCT) for in vitro angiogenesis and invasion assays, xenograft tumor analysis of invasiveness and cell loss scores, and CSC-growth inhibition experiments; two-way ANOVA and Tukey-MCT for apoptosis gene array data; two-way repeated-measures ANOVA and Tukey-MCT for xenograft tumor growth. A P<0.05 was considered statistically significant.

## Supporting Information

Figure S1 **Representative phosphoproteomic analysis of ligand-specific DEspR-signaling pathways.** (A) Human chromosome 4 map with *Dear* (DEspR) location notated, along with R1^88 bp^, reverse primer for DEspR-specific 1^st^ strand cDNA synthesis which also serves as reverse primer for 88 bp amplicon, F1, forward primer for 88 bp amplicon. (B) Comparative binding affinity of anti-hDEspR mAb candidates (open symbols) as the basis for selecting 7c5b2 (red diamond). The other high binding candidates (blue circle, purple triangle) did not grow well. (C) Representative phosphoprotein fluorescent readout of DEspR-signaling proteins activated by VEGFsp and ET1 respectively upon stimulation of DEspR+ Cos1 cell-transfectants at t-30 minutes. Red, VEGFsp-induced or ET1-induced activation of signaling phosphoproteins; blue, non-stimulated DEspR+ Cos1 cell-transfectants serving as reference controls. Phosphoproteins tested in duplicate; GenBank gene names listed; phosphorylated amino acids listed in superscript.
(PDF)

Figure S2 **Increased DEspR+ expression in pancreatic ductal adenocarcinoma and glioblastoma tumor biopsy cores.** Identical exposure settings were used validating comparison of normal pancreas with pancreatic cancer sections, and glioblastoma with normal brain sections respectively; DAPI nuclear stain (blue). (A) Representative low power field (LPF, 200×) and high field (400×) power immunofluorescence images of DEspR+ immunostaining (red) comparing normal pancreas and pancreatic cancer tumor biopsy cores. Bar, 50 microns (200×), 20 microns (400×). Increased DEspR+ expression detected in tumor cells. (B) Representative LPF-200× and HPF-400× immunofluorescence images of DEspR+ immunostaining (red) comparing normal brain and glioblastoma tumor biopsy cores. Bar, 50-microns (200×), 20-microns (400×). (C) Bar Graph of auto exposure times at identical photomicroscopy settings (linear, non-adjusted) representative of immunofluorescence intensity levels (exposure setting≈1/intensity) detected in normal pancreas and DEspR-negative tumor biopsy section biopsy cores vs DEspR+ pancreatic cancer tumor biopsy sections. ANOVA with Tukey's multiple comparisons test, ***, P<0.0001. Black, LPF-200X; Red, HPF-400X fluorescence analysis. (D) Bar Graph of auto exposure times at identical photomicroscopy settings (linear, non-adjusted) representative of immunofluorescence intensity levels (exposure setting≈1/intensity) detected in normal brain and DEspR-negative tumor biopsy section biopsy cores (hatched) vs DEspR+ glioblastoma tumor biopsy sections. ANOVA with Tukey's multiple comparisons test, ***, P<0.0001, **, P<0.001. Black, LPF-200X; Blue, HPF-400X fluorescence analysis.
(PDF)

Figure S3 **Anti-ratDEspR pAb inhibits VEGFsp-induced and ET1-induced angiogenic sprouting in a rat aortic ring assay.** (A) Representative image of aortic ring neovessels in serum; **, ***, ****, secondary, tertiary and quaternary branching; arrow indicates a polygon formed by interconnecting neovessels. (B) Representative image of aortic ring neovessels induced by VEGFsp. (C) Representative image of aortic ring neovessels induced by ET1. (D) Number of neovessels sprouting from rat aortic ring is increased by VEGFsp and ET1 compared to serum and reduced by anti-ratDEspR pAb treatment. One way ANOVA P = 0.0002; Bonferroni's specific pairwise comparison:**, P<0.001; *, P<0.01. (E) Rat aortic ring analysis of angiogenesis measured as # branches per aortic ring. *, P<0.05; VEGFsp, signal peptide for VEGF; ET1,.Endothelin-1.
(PDF)

Figure S4 **Apoptosis gene-pathways affected by DEspR-inhibition in Panc1 and U87-CSCs (GenBank nomenclature) and ARMS detection of spliced DEspR-RNA transcript.** (A) Real-time qPCR analysis of changes in apoptosis pathway genes after 16-hours of DEspR-inhibition of Panc1-CSCs and U87-CSCs. Black arrows, gene changes that promote apoptosis; white arrows, gene changes that decrease apoptosis. Two-way ANOVA with Tukey-MCT: *, P<0.05; **, P<0.01; ***, P<0.001; ****, P<10^−4^; ****** P<10^−6^. (B) Diagram (not to scale) shows relative location of 36 nucleotide (nt-long) reverse primer for cDNA synthesis (R_cDNA_), which also serves as the reverse primer for PCR amplification, and 22-nt-long forward ARMS primer (F_ARMS_) that spans the exon 1-to-exon 2 junction. Successful ARMS should detect a predicted 270 bp PCR amplicon of spliced DEspR-RNA. No unspliced DEspR-RNA, predicted size 342-bp amplicon, should be detected based on the 4 nucleotide discrepancy shown in C. (C) ARMS forward primer 22 nucleotide sequence is specific for spliced exon 1-to-exon 2 junction producing a 270 bp amplicon. F_ARMS_ forward primer is 4 nucleotides discrepant with unspliced DEspR exon 1-intron junction, hence will not produce the predicted size 342 bp PCR amplicon. ARMS assays can detect single nucleotide substitutions; hence 4 nucleotides discrepancy has increased robustness in accuracy and specificity.
(PDF)

Figure S5 **Representative histological and immunofluorescence micrographs of Panc1- CSC nude rat xenograft tumors.** (A) H&E stained section showing tumor cells with high mitotic count per HPF in Panc1-CSC xenograft tumor; (arrows, mitotic cells). (B) Representative merged DEspR+ (red) immunostaining with DIC (differential interference contrast) overlay showing tumor cell heterogeneity with areas of DEspR+ immunostaining close to DEspR+ microvessels and areas of DEspR(-) tumor cells. (C) Human-specific DEspR+ (red) immunostaining of tumor cells at xenograft tumor edge and DEspR+ adjacent microvessels deriving from human Panc1-CSCs. DIC overlay shows refractive red blood cells within the lumen, indicating connectivity of human-specific DEspR+ Panc1 CSC-derived xenograft tumor blood vessels (white arrows, microvessels with rbcs). (D) Rat-specific anti-DEspR mAb does not immunostain intratumoral vasculature or tumor cells of Panc1-CSC xenograft tumors. RBCs within tumor blood vessels indicate connectivity to host rat circulation. White asterisks, micro-vessel lumen with and without RBCs; bar, 20-microns.
(PDF)

Figure S6 Representative histological and immunofluorescence micrographs of U87-CSC nude rat xenograft tumors (untreated A-D, anti-DEspR treated E). (A) H&E stained section showing invasive U87-CSC xenograft tumor cell cluster (black arrows) encroaching onto the subjacent skeletal muscle. Bar, 10-microns. (B) Merged immunofluorescence with DIC overlay showing human-specific 7c5b2 DEspR+ (red) immunostaining of invasive tumor cells at the tumor edge encroaching into the fibrous capsule, and DEspR+ microvessels with red blood cells in the microvessel lumen (white asterisks). Bar, 20-microns. (C) Merged immunofluorescence with control IgG2b isotype-AF468 showing non-specific red fluorescence in red blood cells (RBCs). Bar, 20-microns. (D) H&E stained section showing thin-walled microvessels (yellow arrows) with RBC-filled lumen (red immunofluorescence) surrounded by tumor cells and stromal cells. Bar, 10-microns. (E) Masson-trichrome stained section of anti-DEspR treated xenograft tumor showing tumor cell ‘island’ circumscribed by collagen and close to a microvessel with stabilized wall architecture. Bar, 10 microns.
(PDF)

Table S1 **Comparative analysis of signaling pathways activated upon ET1- and VEGFsp-specific stimulation of hDEspR in DEspR+Cos1 cell-transfectants.**
(DOCX)

Text S1 References for Table S1.
(DOCX)
