# Correction: DEspR Roles in Tumor Vasculo-Angiogenesis, Invasiveness, CSC-Survival and Anoikis Resistance: A ‘Common Receptor Coordinator’ Paradigm

**DOI:** 10.1371/journal.pone.0112335

**Published:** 2014-10-27

**Authors:** The PLOS ONE Staff

There is an error in Figure 1, “DEspR expression in Cos1 cell transfectants, HUVECs and CSCs.” Please see the corrected Figure 1 here.

**Figure 1:**
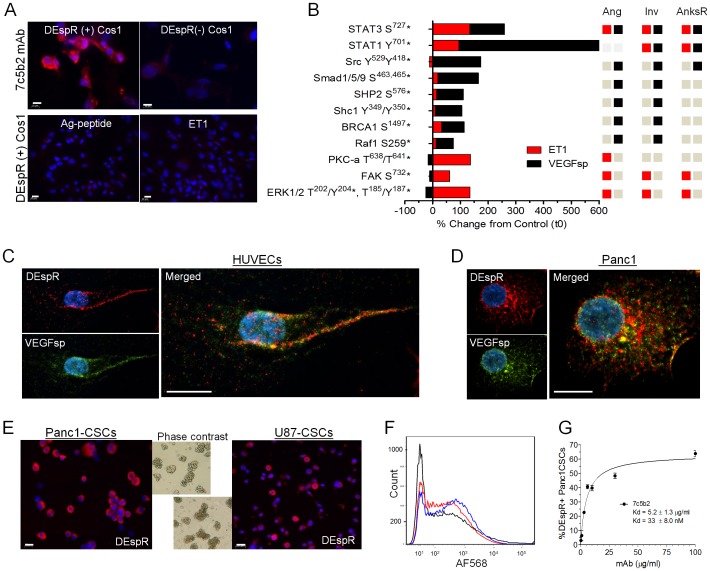
DEspR expression in Cos1 cell transfectants, HUVECs and CSCs. (A) Immunostaining with 7c5b2-mAb detects DEspR expression in Cos1cell DEspR-positive transfectants (DEspR+Cos1), but none in mock-transfected DEspR-negative Cos1cell controls, (DEspR(-)Cos1). Anti-hDEspR 7c5b2-binding on DEspR+Cos1cell-transfectants is displaced by 100X antigenic peptide (Ag-peptide) and by 100X endothelin-1 (ET1). ELISA of 7c5b2 binding to Ag-peptide in Fig. S1B. (B) Bar graph summary of signaling phosphoproteins with greater than 50% change from control (CFC) detected on phosphoproteomic analysis upon VEGFsp-DEspR (black bars) and ET1-DEspR (red bars) activation in DEspR+ Cos1 cell transfectants; see Figure S1C for representative phosphoprotein fluorescent image. Role classification for angiogenesis, invasiveness and anoikis resistance presented based on PubMed publications. Red square, ET1-DEspR activation of phosphoprotein; black square, VEGFsp-DEspR activation of phosphoproteins; gray square, no reports of involvement in said pathway. (C) Confocal photomicroscopy of DEspR (red) and VEGFsp (green) localization and co-localization in endothelial cells (HUVECs) and (D) PDAC Panc1 cells using 7c5b2 anti-hDEspR mAb and anti-VEGFsp polyclonal antibody. DAPI, blue; bar, 10 microns. (E) Representative immunofluorescence images of anchorage-independent PDAC Panc1-CSC and glioblastoma U87-CSC singlets and spheroids after dispersal by mechanical trituration. DEspR+ immunostaining (red); DAPI nuclear stain (blue); bar, 20 microns. Phase contrast images of spheroids ≥50 microns. (F) Superimposed composite of competition FACs analysis of anti-hDEspR 7c5b2 mAb binding to Panc1-CSCs alone (blue), and when displaced by the addition of DEspR ligands at 50×: VEGFsp (black), ET1 (red) respectively. (G) Binding affinity of anti-hDEspR 7c5b2 to Panc1-CSCs showing Kd  =  33±8.0 nM.

The images for Figures 2 and 3 are incorrectly switched. The image that appears as Figure 2 should be Figure 3, and the image that appears as Figure 3 should be Figure 2. The figure legends appear in the correct order. Please see the corrected Figure 2 and Figure 3 here.

**Figure 2:**
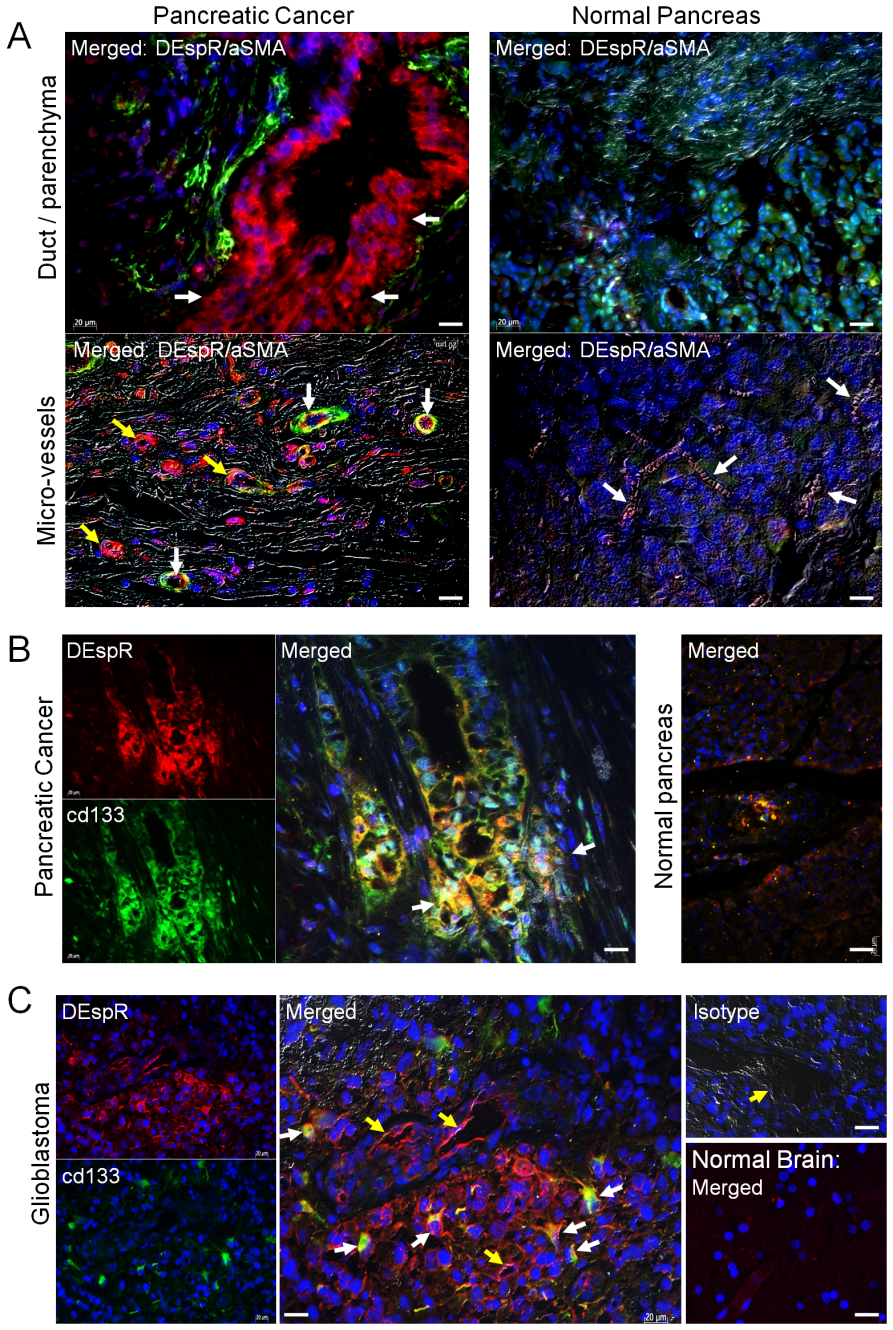
DEspR expression in human pancreatic ductal adenocarcinoma (PDAC) and glioblastoma (GBM). (A) Merged immunofluorescence image of Gr. IV PDAC tumor tissue showing DEspR+ expression (red) in PDAC ductal tumor cells (white arrows) and alpha smooth muscle actin (aSMA)+ expression (green) in adjacent stromal tissue (upper left). DEspR+ and aSMA+ expression and co-expression (yellow) in PDAC tumor microvessels (lower left). Merged immunofluorescence images of normal pancreas detects no DEspR expression in ducts, parenchymal cells (upper right) nor in microvessels (white arrows, lower right). Differential interference contrast (DIC) overlays showing clusters of DEspR+ (red) microvessels (yellow arrows) and co-expressed DEspR+/aSMA+ (yellow) in microvessels (white arrows) within stromal fibrosis area (lower left) in pancreatic cancer but none in normal tissue. (B) Individual and merged immunofluorescence images of DEspR (red), CD133 (green) and double DEspR+/CD133+ immunofluorescence (yellow) shows DEspR+,CD133+ co-expression (yellow) in pancreatic cancer cells at invasive tumor edge (white arrows). Normal pancreatic tissue does not exhibit DEspR+ and CD133+ immunostaining. (C) DEspR+ (red) GBM tumor cells and microvascular endothelium at tumor edge with CD133+ (green) putative CSCs at GBM tumor edge. Merged DEspR+/CD133+ co-expression (yellow-green or yellow) in GBM putative CSCs (white arrows) close to DEspR+ microvessels (yellow arrows). AF568-labeled isotype control (Isotype) shows negative immunofluorescence of GBM section at identical experimental settings; microvessel (yellow arrow). No DEspR+ expression detected in normal brain section. Tumor biopsy core analysis in Figure S2. Bar, 20 microns.

**Figure 3:**
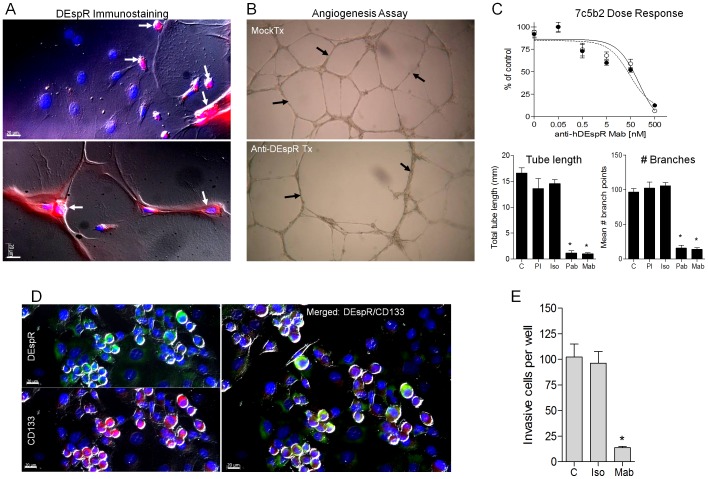
In vitro analysis of DEspR roles. (A) DEspR-positive (red) immunostaining of HUVECs undergoing angiogenic ‘tube’ formation (white arrows) in contrast to DEspR-negative quiescent HUVECs (DAPI-stained blue nuclei). DEspR+ expression in HUVECs forming angiogenic networks. (B) Representative HUVECs angiogenic network formation in Matrigel in control mock-treated angiogenic conditions (Mock Tx) in contrast to decreased angiogenic networks upon DEspR-inhibition (Anti-DEspR Tx). (C) Dose response curve of anti-hDEspR(7c5b2) mAb inhibition of angiogenesis measured as tube length (black circle) and number of branch points (open circle). DEspR-inhibition at 500 nM dose of total angiogenic tube length (Tube length) and mean number of branch points (# Branches) comparing control untreated (C), pre-immune negative control (PI) for polyclonal anti-hDEspR ab (Pab), isotype negative control (Iso) and for anti-hDEspR 7c5b2 (Mab). Anti-angiogenic effects in rat aortic ring assays (Figure S3). (D) Representative immunofluorescence analysis of nonCSC adherent Panc1-cells; DEspR-positive (green) immunostaining (DEspR), CSC-marker CD133-positive immunostaining (red). Merged DEspR and CD133 immunostaining with differential interference contrast (DIC) overlay showing co-expression in subset of Panc1-cells (yellow-green) forming clusters mounding above the culture-dish plane; DAPI nuclear stain (blue). (E) Anti-hDEspR 7c5b2 inhibition of Panc1 cell invasiveness; C, non-treated cells, Iso, isotype IgG2b mock-treated cells, Mab, anti-hDEspR 7c5b2 mAb at 500 nM. *, P<0.0001 one-way ANOVA with Tukey multiple comparisons testing of anti-DEspR antibodies with respective controls; bar, 20 microns.
