# Supplementary figures and images for: DEspR Roles in Tumor Vasculo-Angiogenesis, Invasiveness, CSC-Survival and Anoikis Resistance: A ‘Common Receptor Coordinator’ Paradigm
Source: PLoS One. 2014 Jan 21;9(1):e85821. doi: 10.1371/journal.pone.0085821 (PMC3897535; doi:10.1371/journal.pone.0085821)

A

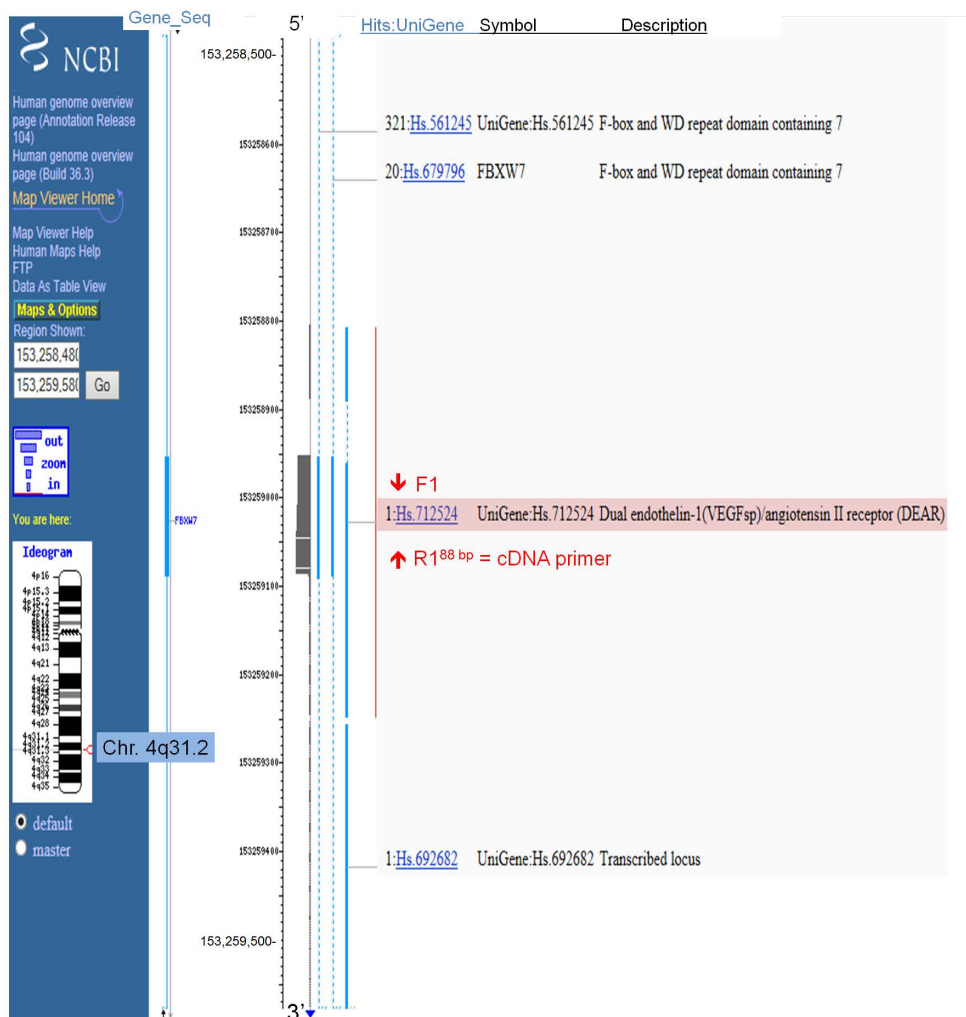

B

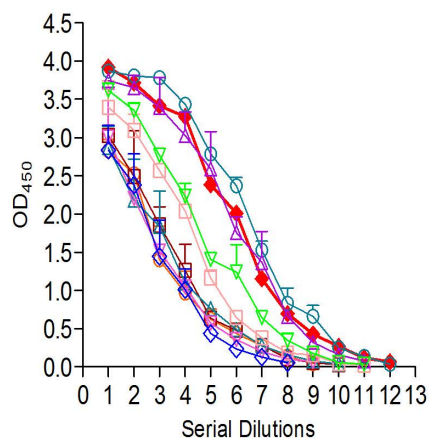

C

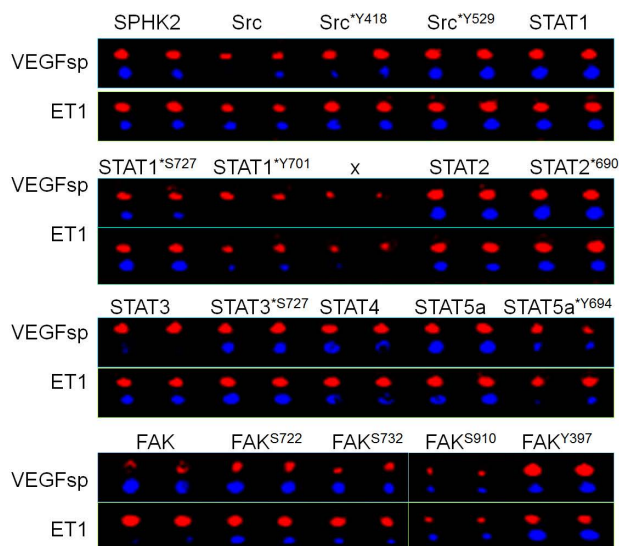

Supplement: Figure S1 — Representative phosphoproteomic analysis of ligand-specific DEspR-signaling pathways. (A) Human chromosome 4 map with Dear (DEspR) location notated, along with R188 bp, reverse primer for DEspR-specific 1st strand cDNA synthesis which also serves as reverse primer for 88 bp amplicon, F1, forward primer for 88 bp amplicon. (B) Comparative binding affinity of anti-hDEspR mAb candidates (open symbols) as the basis for selecting 7c5b2 (red diamond). The other high binding candidates (blue circle, purple triangle) did not grow well. (C) Representative phosphoprotein fluorescent readout of DEspR-signaling proteins activated by VEGFsp and ET1 respectively upon stimulation of DEspR+ Cos1 cell-transfectants at t-30 minutes. Red, VEGFsp-induced or ET1-induced activation of signaling phosphoproteins; blue, non-stimulated DEspR+ Cos1 cell-transfectants serving as reference controls. Phosphoproteins tested in duplicate; GenBank gene names listed; phosphorylated amino acids listed in superscript. (PDF) [file pone.0085821.s001.pdf]

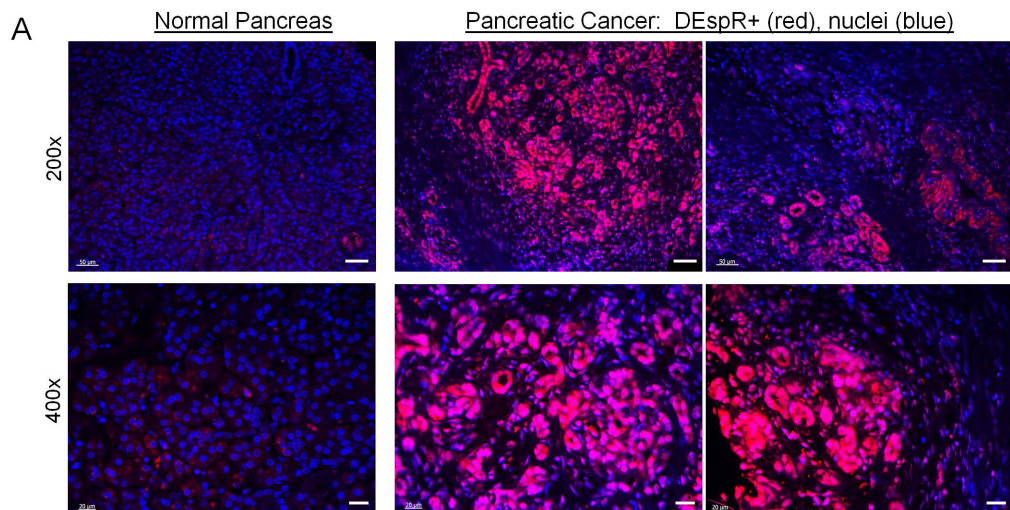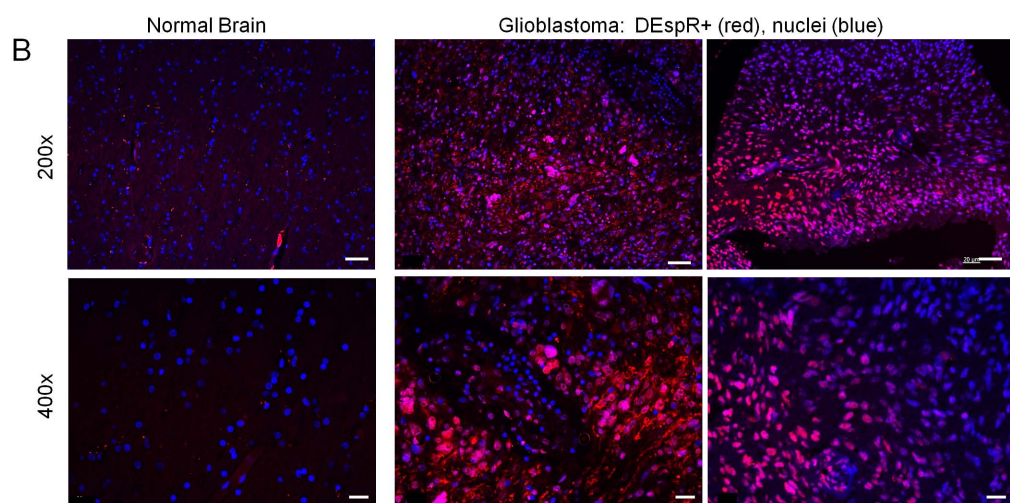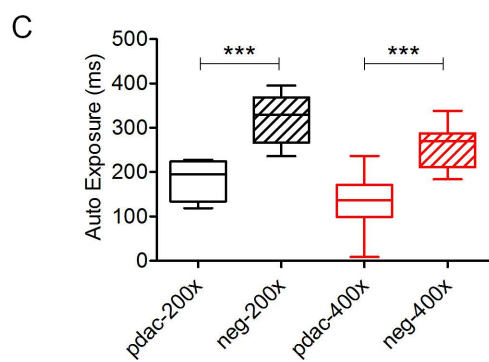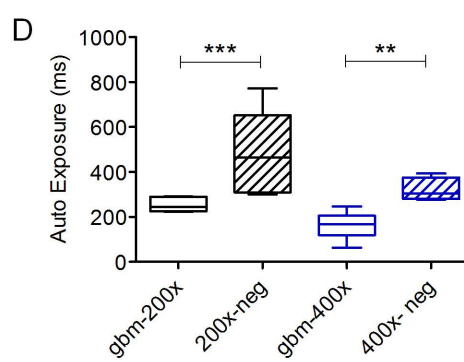

Supplement: Figure S2 — Increased DEspR+ expression in pancreatic ductal adenocarcinoma and glioblastoma tumor biopsy cores. Identical exposure settings were used validating comparison of normal pancreas with pancreatic cancer sections, and glioblastoma with normal brain sections respectively; DAPI nuclear stain (blue). (A) Representative low power field (LPF, 200×) and high field (400×) power immunofluorescence images of DEspR+ immunostaining (red) comparing normal pancreas and pancreatic cancer tumor biopsy cores. Bar, 50 microns (200×), 20 microns (400×). Increased DEspR+ expression detected in tumor cells. (B) Representative LPF-200× and HPF-400× immunofluorescence images of DEspR+ immunostaining (red) comparing normal brain and glioblastoma tumor biopsy cores. Bar, 50-microns (200×), 20-microns (400×). (C) Bar Graph of auto exposure times at identical photomicroscopy settings (linear, non-adjusted) representative of immunofluorescence intensity levels (exposure setting≈1/intensity) detected in normal pancreas and DEspR-negative tumor biopsy section biopsy cores vs DEspR+ pancreatic cancer tumor biopsy sections. ANOVA with Tukey's multiple comparisons test, ***, P<0.0001. Black, LPF-200X; Red, HPF-400X fluorescence analysis. (D) Bar Graph of auto exposure times at identical photomicroscopy settings (linear, non-adjusted) representative of immunofluorescence intensity levels (exposure setting≈1/intensity) detected in normal brain and DEspR-negative tumor biopsy section biopsy cores (hatched) vs DEspR+ glioblastoma tumor biopsy sections. ANOVA with Tukey's multiple comparisons test, ***, P<0.0001, **, P<0.001. Black, LPF-200X; Blue, HPF-400X fluorescence analysis. (PDF) [file pone.0085821.s002.pdf]

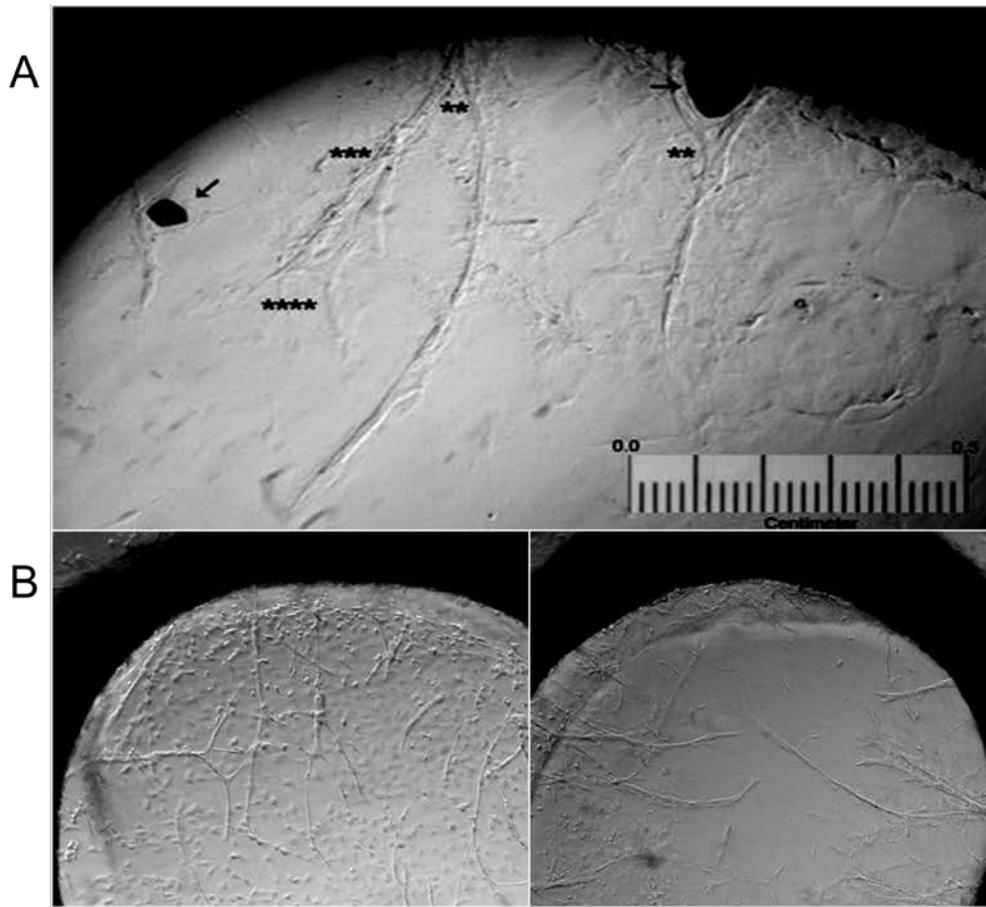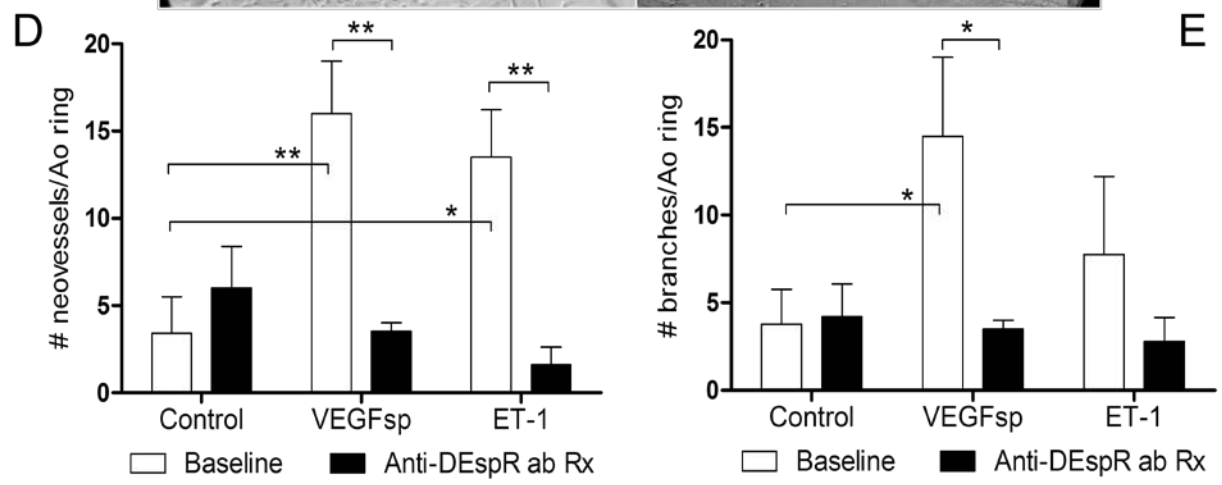

Supplement: Figure S3 — Anti-ratDEspR pAb inhibits VEGFsp-induced and ET1-induced angiogenic sprouting in a rat aortic ring assay. (A) Representative image of aortic ring neovessels in serum; **, ***, ****, secondary, tertiary and quaternary branching; arrow indicates a polygon formed by interconnecting neovessels. (B) Representative image of aortic ring neovessels induced by VEGFsp. (C) Representative image of aortic ring neovessels induced by ET1. (D) Number of neovessels sprouting from rat aortic ring is increased by VEGFsp and ET1 compared to serum and reduced by anti-ratDEspR pAb treatment. One way ANOVA P = 0.0002; Bonferroni's specific pairwise comparison:**, P<0.001; *, P<0.01. (E) Rat aortic ring analysis of angiogenesis measured as # branches per aortic ring. *, P<0.05; VEGFsp, signal peptide for VEGF; ET1,.Endothelin-1. (PDF) [file pone.0085821.s003.pdf]

A.

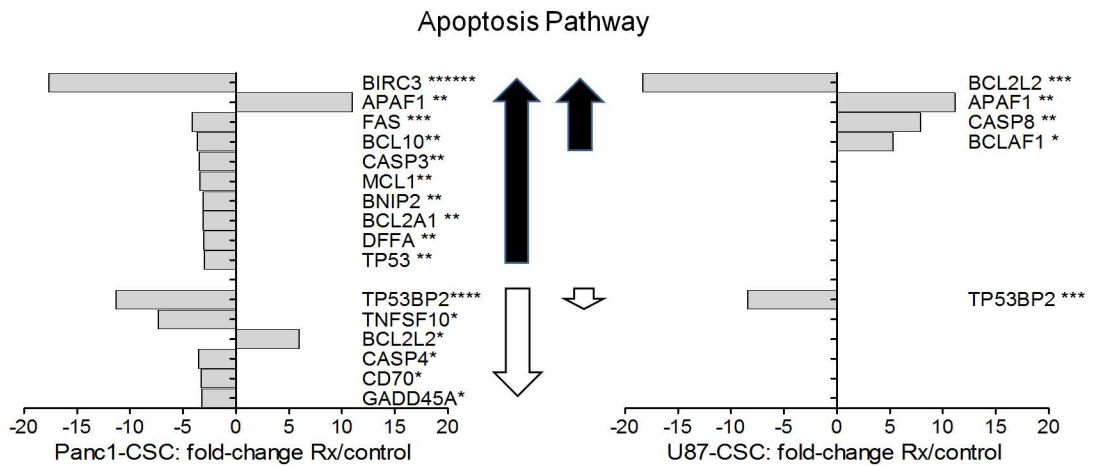

B.

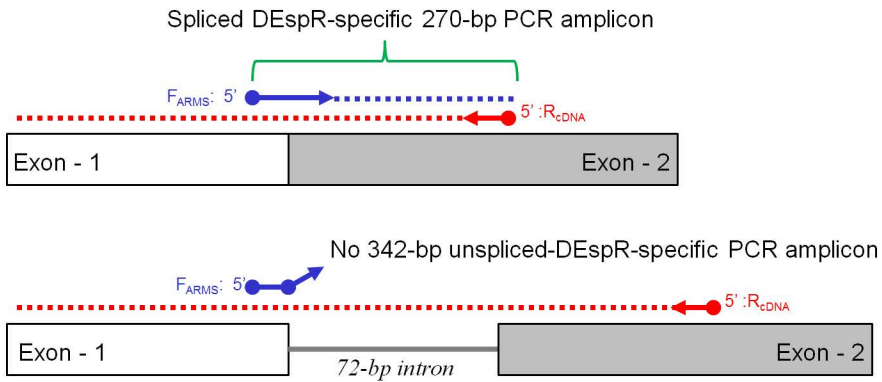

C.

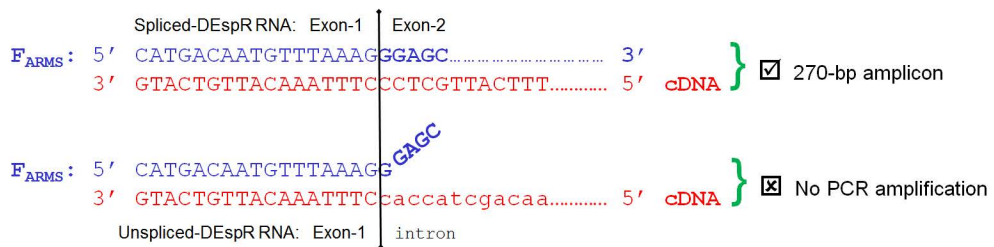

Supplement: Figure S4 — Apoptosis gene-pathways affected by DEspR-inhibition in Panc1 and U87-CSCs (GenBank nomenclature) and ARMS detection of spliced DEspR-RNA transcript. (A) Real-time qPCR analysis of changes in apoptosis pathway genes after 16-hours of DEspR-inhibition of Panc1-CSCs and U87-CSCs. Black arrows, gene changes that promote apoptosis; white arrows, gene changes that decrease apoptosis. Two-way ANOVA with Tukey-MCT: *, P<0.05; **, P<0.01; ***, P<0.001; ****, P<10−4; ****** P<10−6. (B) Diagram (not to scale) shows relative location of 36 nucleotide (nt-long) reverse primer for cDNA synthesis (RcDNA), which also serves as the reverse primer for PCR amplification, and 22-nt-long forward ARMS primer (FARMS) that spans the exon 1-to-exon 2 junction. Successful ARMS should detect a predicted 270 bp PCR amplicon of spliced DEspR-RNA. No unspliced DEspR-RNA, predicted size 342-bp amplicon, should be detected based on the 4 nucleotide discrepancy shown in C. (C) ARMS forward primer 22 nucleotide sequence is specific for spliced exon 1-to-exon 2 junction producing a 270 bp amplicon. FARMS forward primer is 4 nucleotides discrepant with unspliced DEspR exon 1-intron junction, hence will not produce the predicted size 342 bp PCR amplicon. ARMS assays can detect single nucleotide substitutions; hence 4 nucleotides discrepancy has increased robustness in accuracy and specificity. (PDF) [file pone.0085821.s004.pdf]

A

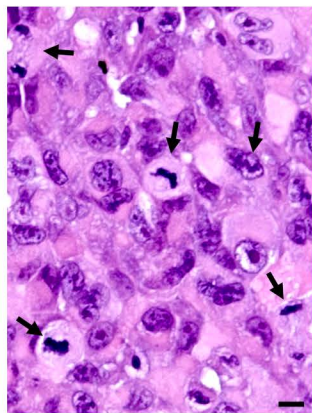

B

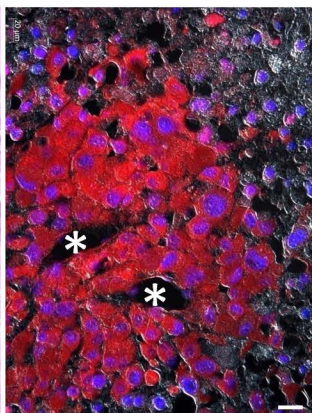

C

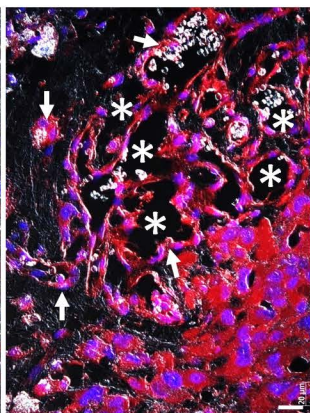

D

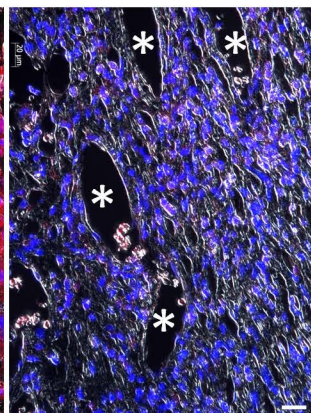

Supplement: Figure S5 — Representative histological and immunofluorescence micrographs of Panc1- CSC nude rat xenograft tumors. (A) H&E stained section showing tumor cells with high mitotic count per HPF in Panc1-CSC xenograft tumor; (arrows, mitotic cells). (B) Representative merged DEspR+ (red) immunostaining with DIC (differential interference contrast) overlay showing tumor cell heterogeneity with areas of DEspR+ immunostaining close to DEspR+ microvessels and areas of DEspR(-) tumor cells. (C) Human-specific DEspR+ (red) immunostaining of tumor cells at xenograft tumor edge and DEspR+ adjacent microvessels deriving from human Panc1-CSCs. DIC overlay shows refractive red blood cells within the lumen, indicating connectivity of human-specific DEspR+ Panc1 CSC-derived xenograft tumor blood vessels (white arrows, microvessels with rbcs). (D) Rat-specific anti-DEspR mAb does not immunostain intratumoral vasculature or tumor cells of Panc1-CSC xenograft tumors. RBCs within tumor blood vessels indicate connectivity to host rat circulation. White asterisks, micro-vessel lumen with and without RBCs; bar, 20-microns. (PDF) [file pone.0085821.s005.pdf]

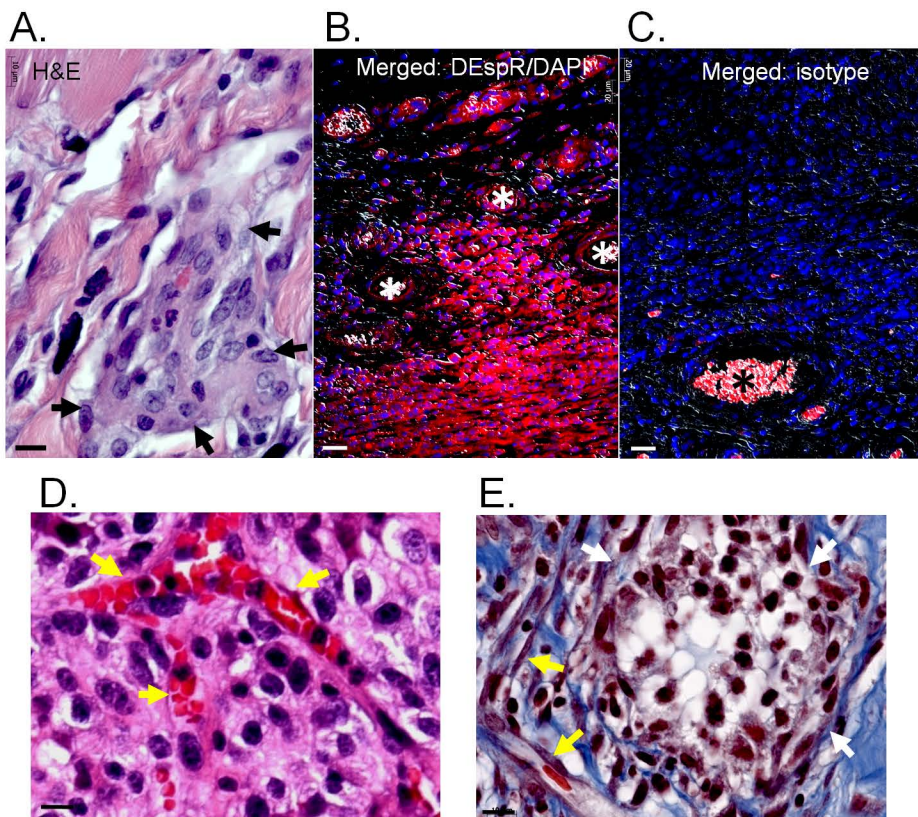

Supplement: Figure S6 — Representative histological and immunofluorescence micrographs of U87-CSC nude rat xenograft tumors (untreated A-D, anti-DEspR treated E). (A) H&E stained section showing invasive U87-CSC xenograft tumor cell cluster (black arrows) encroaching onto the subjacent skeletal muscle. Bar, 10-microns. (B) Merged immunofluorescence with DIC overlay showing human-specific 7c5b2 DEspR+ (red) immunostaining of invasive tumor cells at the tumor edge encroaching into the fibrous capsule, and DEspR+ microvessels with red blood cells in the microvessel lumen (white asterisks). Bar, 20-microns. (C) Merged immunofluorescence with control IgG2b isotype-AF468 showing non-specific red fluorescence in red blood cells (RBCs). Bar, 20-microns. (D) H&E stained section showing thin-walled microvessels (yellow arrows) with RBC-filled lumen (red immunofluorescence) surrounded by tumor cells and stromal cells. Bar, 10-microns. (E) Masson-trichrome stained section of anti-DEspR treated xenograft tumor showing tumor cell ‘island’ circumscribed by collagen and close to a microvessel with stabilized wall architecture. Bar, 10 microns. (PDF) [file pone.0085821.s006.pdf]
