# Supplementary material for: DEspR Roles in Tumor Vasculo-Angiogenesis, Invasiveness, CSC-Survival and Anoikis Resistance: A ‘Common Receptor Coordinator’ Paradigm
Source: PLoS One. 2014 Jan 21;9(1):e85821. doi: 10.1371/journal.pone.0085821 (PMC3897535; doi:10.1371/journal.pone.0085821)
Supplement: Table S1 — Comparative analysis of signaling pathways activated upon ET1- and VEGFsp-specific stimulation of hDEspR in DEspR+Cos1 cell-transfectants. (DOCX) [file pone.0085821.s007.docx]

| **Table S1. Comparative analysis of signaling pathways activated upon ET1- and VEGFsp-specific stimulation of hDEspR in DEspR+Cos1 cell-transfectants.** | | | | | | | |  |
| --- | --- | --- | --- | --- | --- | --- | --- | --- |
| **Protein Name** | **Symbol** | **P*-Site** | **ET1**  **(%CFC)** | **VEGFsp**  **(%CFC)** | **Pro-**  **Angiogenesis** | **Pro-**  **Cancer** | **Pro-**  **Stem cell** | **Anoikis Resistance** |
| Breast cancer type 1 susceptibility protein | BRCA1 | S1497 | **32** | **82** | (Kang et al., 2006) | (Hesling et al., 2004) |  |  |
| Cyclin-dependent protein-serine kinase ½ | CDK1/2 | T14/Y15 | **53** | *-16* |  | (Johnson et al., 2009; Johnson et al., 2010) |  |  |
|  |  | Y15 | **281** | -57 |  |  |  |  |
| Extracellular regulated protein-serine kinase 1/2 (p44/p42 MAP kinases) | ERK1/2 | T202+Y204;  T185+Y187 | **135** | *-25* | (Langenfeld et al., 2005; Xu et al., 2008) | (Balmano and Cook 2009; Fremin and Meloche 2010; Gollob et al., 2006) | (Morishita et al., 2007; Wang et al., 2010) |  |
| Focal adhesion protein-tyrosine kinase | FAK | S722 | **55** | *-38* | (Vadali et al., 2007; Luo and Guan 2010) | (Luo and Guan 2010; Provenzano et al., 2008) | (Morishita et al., 2007; Luo and Guan 2010) |  |
|  |  | S732 | **62** | *-11* |  |  |  |  |
|  |  | Panspecific | **205** | 0 |  |  |  |  |
| Hepatocyte growth factor receptor-tyrosine kinase | Met | Panspecific | **384** | 0 | (Fan et al., 2000; Colombo et al., 2007; Matsumoto and Nakamura 2008) | (Matsumoto and Nakamura 2008; Ma et al., 2007) | (Yang et al., 2007) |  |
| Proliferating cell nuclear antigen | PCNA | Panspecific | *-47* | **119** |  | (Stuart-Harris et al., 2008) |  |  |
| Protein-serine kinase C-alpha | PKCa | T638/T641 | **137** | *-17* | (Wellner et al., 1999; Xu et al., 2008) |  |  |  |
| Protein-serine kinase C-epsilon | PKCe | Panspecific | **103** | *-29* | (Davis et al., 2007; Yamamura et al., 1996; Gardner and Olah 2003; Heidkamp et al., 2003) | (Heidkamp et al., 2003) | (Morishita et al., 2007; Heidkamp et al., 2003) |  |
| Raf1 proto-oncogene-encoded protein-serine kinase | Raf1 | S259 | 12 | **63** | (Malecki et al., 2004) | (Hoogwater et al., 2010) |  |  |
| SH2 domain-containing transforming protein 1 | Shc1 | Y349, Y350 | 9 | **97** | (Ursini-Siegel et al., 2008; Audero et al., 2004; Saucier et al., 2004) | (Ursini-Siegel et al., 2008; Northey et al., 2008; Saucier et al., 2002) |  |  |
| Protein-tyrosine phosphatase 1D | SHP2 | S576 | 14 | **97** | (Agazie et al., 2003; Chernock et al., 2001; Marron et al., 2000) | (Agazie et al., 2003; Zhou et al., 2008; Zhou et al., 2009) | (Zhou et al., 2008; Hagihara et al., 2009; Wu et al., 2009; Ke et al., 2007) |  |
| SMA- and mothers against decapentaplegic homologs 1/5/9 | Smad 1/5/9 | S463+S465/  S465+S467 | 18 | **147** | (Langenfeld et al., 2005) | (Liu et al., 2009) | (Blank et al., 2008) |  |
| Src proto-oncogene-encoded protein-tyrosine kinase | Src | Y529 | -20 | **73** | (Davis et al., 2007) | (Johnson and Gallick 2007; Lin et al., 2004; Mezquita et al., 2010) |  | (Diaz-Montero et al., 2006; Sakuma et al., 2010) |
|  |  | Y418 | -11 | **174** |  |  |  |  |
| Signal transducer and activator of transcription 1 | STAT1 | S727 | **86** | **123** | (Schultz et al., 2010; Heuser and Humphries 2010) | (Schultz et al., 2010) | (Heuser and Humphries 2010) | (Chen et al., 2011) |
|  |  | Y701 | **95** | **557** |  |  |  |  |
| Signal transducer and activator of transcription 3 | STAT3 | S727 | **133** | **126** | (Jung et al., 2005; Jarnicki et al., 2010; Yu et al., 2009) | (Jarnicki et al., 2010; Yu et al., 2009) | (Covey and Levison 2007) | (Du et al., 2009) |
| hDEspR, human dual endothelin-1/vascular endothelial growth factor-signal peptide receptor; ET1, endothelin 1; VEGFsp, vascular endothelial growth factor-signal peptide; % CFC, percentage change in treated vs non-treated control averages: %CFC = [Treated – Control]/Control ave] x 100. Phospho-site, phosphorylation site detected with phosphorylated site-specific antibodies. Data represent >50% CFC taken from mean of treated vs control non-treated duplicates with % error range <20%. %error range = [Treated _A_ – ave]/ave x 100. Kinexus antibody array: phosphoprotein-specific ab to detect phosphorylation changes, and panspecific antibodies to detect expression changes. | | | | | | | | |
