# Supplementary material for: DEspR Roles in Tumor Vasculo-Angiogenesis, Invasiveness, CSC-Survival and Anoikis Resistance: A ‘Common Receptor Coordinator’ Paradigm
Source: PLoS One. 2014 Jan 21;9(1):e85821. doi: 10.1371/journal.pone.0085821 (PMC3897535; doi:10.1371/journal.pone.0085821)
Supplement: Text S1 — References for Table S1. (DOCX) [file pone.0085821.s008.docx]

**References**

Agazie YM, Movilla N, Ischenko I, Hayman MJ (2003) The phosphotyrosine phosphatase SHP2 is a critical mediator of transformation induced by the oncogenic fibroblast growth factor receptor 3. Oncogene 22: 6909-6918.

Audero E, Cascone I, Maniero F, Napione L, Arese M, Lanfrancone L, Bussolino F (2004) Adaptor ShcA protein binds tyrosine kinase Tie2 receptor and regulates migration and sprouting but not survival of endothelial cells. J Biol Chem 279: 13224-13233.

Balmano K, Cook SJ (2009) Tumour cell survival signaling by the ERK1/2 pathway. Cell Death Differ 16: 368-377.

Blank U, Karlsson G, Karlsson S (2008) Signaling pathways governing stem-cell fate. Blood 111: 494-503.

Chernock RD, Cherla RP, Ganju RK (2001) SHP2 and cbl participate in alpha-chemokine receptor CXCR4-mediated signaling pathways. Blood 97: 608-615.

Chen Z, Ma X, Zhang H, Sun X, Shen S, Li Y, Gu Y, Wang Y, Yan S, Yu Q (2011) Negative regulation of interferon-gamma/STAT1 signaling through cell adhesion and cell density-dependent STAT1 dephosphorylation. Cell Signal 23: 1404-1412.

Colombo ES, Menicucci G, McGuire PG, Das A (2007) Hepatocyte growth factor/scatter factor promotes retinal angiogenesis through increased urokinase expression. Invest. Ophthalmol Vis Sci 48: 1793-1800.

Covey MV, Levison SW (2007) Leukemia inhibitory factor participates in the expansion of neural stem/progenitors after perinatal hypoxia/ischemia. Nueroscience 148: 501-509.

Davis GE, Koh W, Stratman AN (2007) Mechanisms controlling human endothelial lumen formation and tube assembly in three-dimensional extracellular matrices. Birth Defects Research 81: 270-285.

Diaz-Montero CM, Wygant JN, McIntyre BW (2006) PI3-K/Akt-mediated anoikis resistance of human osteosarcoma cells requires Src activation. Eur J Cancer 42: 1491-1500.

Du XL, Yang H, Liu SG, Luo ML, Hao JJ, Zhang Y, Lin DC, Xu X, Cai Y, Zhan QM, et al. (2009) Calreticulin promotes cell motility and enhances resistance to anoikis through STAT3-CTTN-Akt pathway in esophageal squamous cell carcinoma. Oncogene 28: 3714-3722.

Fan S, Xian Y, Wang JA, Yuan RQ, Meng Q, Cao Y, Laterra JJ, Goldberg ID, Rosen EM (2000) The cytokine hepatocyte growth factor/scatter factor inhibits apoptosis and enhances DNA repair by a common mechanism involving signaling through phosphatidyl inositol 3’kinase. Oncogene 19: 2212-2223.

Fremin C, Meloche S (2010) From basic research to clinical development of MEK1/2 inhibitors for cancer therapy. J Hematol Oncology 3: 8-18.

Gardner AM, Olah ME (2003) Distinct protein kinase C isoforms mediate regulation of vascular endothelial growth factor expression by A2A adenosine receptor activation and phorbol esters in pheochromocytoma PC12 cells. J Biol Chem 278: 15421-15428.

Gollob JA, Wilhelm S, Carter C, Kelley SL (2006) Role of Raf kinase in cancer: therapeutic potential of targeting the Raf/MEK/ERK signal transduction pathway. Semin Oncol 33: 392-406.

Hagihara K, Zhang EE, Ke YH, Liu G, Liu JJ, Rao Y, Feng GS (2009) Shp2 acts downstream of SDF-1alpha/CXCR4 in guiding granule cell migration during cerebellar development. Dev Biol 334: 276-284.

Heidkamp MC, Bayer AL, Scully BT, Eble DM, Samarel AM (2003) Activation of focal adhesion kinase by protein kinase C epsilon in neonatal rat ventricular myocytes. Am J Physiol Heart Circ Physiol 285: H1684-H1696.

Hesling C, D’Incan M, D’Incan C, Souteyrand P, Monboisse JC, Pasco S, Madelmont JC, Bignon YJ (2004) Down regulation of BRCA1 in A375 melanoma cell line increases radio-sensitivity and modifies metastatic and angiogenic gene expression. J Invest Dermatol 122: 369-380.

Heuser M, Humphries RK (2010) Biologic and experimental variation of measured cancer stem cells. Cell Cycle 9: 909-912.

Hoogwater FJ, Nijkamp MW, Smakman N, Steller EJ, Emmink BL, Westendorp BF, Raats DA, Sprick MR, Schaefer U, Van Houdt WJ, et al. (2010) Oncogenic K-Ras turns death receptors into metastasis-promoting receptors in human and mouse colorectal cancer cells. Gastroenterology 138: 2357-2367.

Jarnicki A, Putoczki T, Ernst M (2010) STAT3: linking inflammation to epithelial cancer – more than a “gut” feeling? Cell Division 5: 14doi:10.1186/1747-1028-14

Johnson FM, Gallick GE (2007) SRC family nonreceptor tyrosine kinases as molecular targets for cancer therapy. Anticancer Agents Med Chem 7: 651-659.

Johnson N, Cai D, Kennedy RD, Pathania S, Arora M, Li YC, D’Andrea AD, Parvin JD, Shapiro GI (2009) Cdk1 participates in BRCA1-dependent S phase checkpoint control in response to DNA damage. Mol Cell 35: 327-339.

Johnson N, Bentley J, Wang LZ, Newell DR, Robson CN, Shapiro GI, Curtin NJ (2010) Pre-clinical evaluation of cyclin-dependent kinase 2 and 1 inhibitionin anti-estrogen-sensitive and resistant breast cancer cells. Br J Cancer 102: 342-350.

Jung JE, Lee HG, Cho IH, Chung DH, Yoon SH, Yang YM, Lee JW, Choi S, Park JW, Ye SK, et al. (2005) STAT3 is a potential modulator of HIF-1-mediated VEGF expression in human renal carcinoma cells. FASEB J 19: 1296-1298.

Kang HJ, Kim HJ, Rih JK, Mattson TL, Kim KW, Cho CH, Isaacs JS, Bae I (2006) BRCA1 plays a role in the hypoxic response by regulating HIF-1α stability and by modulating vascular endothelial growth factor expression. J Biol Chem 281: 13047-13056.

Ke Y, Zhang EE, Hagihara K, Wu D, Pang Y, Klein R, Curran T, Ranscht B, Feng GS (2007) Deletion of Shp2 in the brain leads to defective proliferation and differentiation in neural stem cells and early postnatal lethality. Mol Cell Biol 27: 6706-6717.

[Langenfeld EM, Kong Y, Langenfeld J](http://www.ncbi.nlm.nih.gov/pubmed/16380505?ordinalpos=1&itool=EntrezSystem2.PEntrez.Pubmed.Pubmed_ResultsPanel.Pubmed_RVDocSum) (2005) Bone morphogenetic protein-2-induced transformation involves the activation of mammalian target of rapamycin. Mol Cancer Res 3: 679-684.

Lin EH, Hui AY, Meens JA, Tremblay EA, Schaefer E, Elliott BE (2004) Disruption of Ca2+-dependent cell-matrix adhesion enhances c-Src kinase activity, but causes dissociation of the c-Src/FAK complex and dephosphorylation of tyrosine-577 of FAK in carcinoma cells. Exp Cell Res 293: 1-13.

Liu IM, Schilling SH, Knouse KA, Choy L, Derynck R, Wang XF (2009) TGFbeta-stimulated Smad1/5 phosphorylation requires the ALK5 L45 loop and mediates the pro-migratory TGFbeta switch. EMBO J 28: 88-98.

Luo M, Guan JL (2010) Focal adhesion kinase: a prominent determinant in breast cancer initiation, progression and metastasis. Cancer Lett 289: 127-139.

Ma PC, Tretiakova MS, Nallasura V, Jagadeeswaran R, Husain AN, Salgia R (2007) Downstream signaling and specific inhibition of c-MET/HGF pathway in small cell lung cancer: implications for tumour invasion. Br J Cancer 97: 368-377.

Malecki M, Seneta M, Miloszewska J, Trembacz H, Przbyszewska M, Janik P (2004) Role of v-Raf and truncated form RAF1 in the induction of vascular endothelial growth factor and vascularization. Oncol Rep 11: 161-165.

Marron MB, Hughes DP, McCarthy MJ, Beaumont ER, Brindle NP (2000) Tie-1 receptor tyrosine kinase endodomain interaction with SHP2: potential signaling mechanisms and roles in angiogenesis. Adv Exp Med Biol 476: 35-46.

Matsumoto K, Nakamura T (2008) NK4 gene therapy targeting HGF-Met and angiogenesis. Front Biosci 13: 1943-1951.

Mezquita B, Mezquita J, Pau M, Mezquita C (2010) A novel intracellular isoform of VEGFR-1 activates Src and promotes cell invasion in MDA-MB-231 breast cancer cells. J Cell Biochem 110: 732-742.

[Morishita R, Ueda H, Ito H, Takasaki J, Nagata K, Asano T](http://www.ncbi.nlm.nih.gov/pubmed/17707940?ordinalpos=12&itool=EntrezSystem2.PEntrez.Pubmed.Pubmed_ResultsPanel.Pubmed_RVDocSum) (2007) Involvement of Gq/11 in both integrin signal-dependent and -independent pathways regulating endothelin-induced neural progenitor proliferation. Neurosci Res 59: 205-214.

Northey JJ, Chmielecki J, Ngan E, Russo C, Annis MG, Muller WJ, Siegel PM (2008) Signaling through ShcA is required for TGF-beta and Neu/ErbB-2 induced breast cancer cell motility and invasion. Mol Cell Biol 28: 3162-3176.

Provenzano PP, Inman DR, Eliceiri KW, Beggs HE, Keely PJ (2008) Mammary epithelial-specific disruption of focal adhesion kinase retards tumor formation and metastasis in a transgenic mouse model of human breast cancer. Am J Pathol 173: 1551-1565.

Sakuma Y, Takeuchi T, Nakamura Y, Yoshihara M, Matsukuma S, Nakayama H, Ohgane H, Yokose T, Kameda Y, Tsuchiya E, et al. (2010) Lung adenocarcinoma cells floating in lymphatic vessels resist anoikis by expressing phosphorylated Src. J Pathol 220: 574-585.

Saucier C, Papavailiou V, Palazzo A, Naujokas MA, Kremer R, Park M (2002) Use of signal specific receptor tyrosine kinase oncoproteins reveals that pathways downstream from Grb2 or Shc are sufficient for cell transformation and metastasis. Oncogene 21: 1800-1811.

Saucier C, Khoury H, Lai KM, Peschard P, Dankort D, Naujokas MA, Holash J, Yancopoulos GD, Muller WJ, Pawson T, et al. (2004) The Shc adaptor protein is critical for VEGF induction by Met/HGF and Erb B2 receptors and for early onset of tumor angiogenesis. Proc Natl Acad Sci 101: 2345-2350.

Schultz J, Koczan D, Schmitz U, Ibrahim SM, Pilch D, Landsberg J, Kunz M (2010) Tumor-promoting role of signal transducer and activator of transcription (Stat)1 in late-stage melanoma growth. Clin Exp Metastasis 27: 133-140.

Stuart-Harris R, Caldas C, Pinder SE, Pharoah P (2008) Proliferation markers and survival in early breast cancer: a systematic review and meta-analysis of 85 studies in 32,825 patients. Breast 17: 323-334.

Ursini-Siegel J, Hardy WR, Zuo D, Lam SHL, Sanguin-Gendreau V, Cardiff RD, Pawson T, Muller W (2008) ShcA signaling is essential for tumour progression in mouse models of human breast cancer. EMBO J 27: 910-920.

[Vadali K, Cai X, Schaller MD](http://www.ncbi.nlm.nih.gov/pubmed/17968709?ordinalpos=40&itool=EntrezSystem2.PEntrez.Pubmed.Pubmed_ResultsPanel.Pubmed_RVDocSum) (2007) Focal adhesion kinase: an essential kinase in the regulation of cardiovascular functions. IUBMB Life 59: 709-716.

Wang Y, Zhu Y, Qiu F, Zhang T, Chen Z, Zheng S, Huang J (2010) Activation of Akt and MAPK pathways enhances the tumorigenicity of CD133+ primary colon cancer cells. Carcinogenesis 31: 1376-1380.

Wellner M, Maasch C, Kupprion C, Lindschau C, Luft FC, Haller H (1999) The proliferative effect of vascular endothelial growth factor requires protein kinase C-alpha and protein kinase C-zeta. Arterioscler Thromb Vasc Biol 19: 178-185.

Wu D, Pang Y, Ke Y, Yu J, He Z, Tautz L, Mustelin T, Ding S, Huang Z, Feng GS (2009) A conserved mechanism for control of human and mouse embryonic stem cell pluripotency and differentiation by shp2 tyrosine phosphatase. PLoS One 4: e4914.

Xu H, Czerwinski P, Hortmann M, Sohn HY, Forstermann U, Li H (2008) Protein kinase C alpha promotes angiogenic activity of human endothelial cells via induction of vascular endothelial growth factor. Cardiovasc Res 78: 349-355.

Xu J, Liu X, Jiang Y, Chu L, Hao H, Liu Z, Verfaillie C, Zweier J, Gupta K, Liu Z (2008) MAPK/ERK signaling mediates VEGF-induced bone marrow stem cell differentiation into endothelial cell. J Cell Mol Med 12: 2395-2406.

Yamamura S, Nelson PR, Kent KC (1996) Role of protein kinase C in attachment, spreading, and migration of human endothelial cells. J Surg Res 63: 349-354.

[Yang Z, Wang W, Ma D, Zhang Y, Wang L, Zhang Y, Xu S, Chen B, Miao D, Cao K, et al.](http://www.ncbi.nlm.nih.gov/pubmed/17973092?ordinalpos=2&itool=EntrezSystem2.PEntrez.Pubmed.Pubmed_ResultsPanel.Pubmed_RVDocSum) (2007) Recruitment of stem cells by hepatocyte growth factor via intracoronary gene transfection in the postinfarction heart failure. Sci China C Life Sci 50: 748-752.

Yu H, Pardoll D, Jove R (2009) STATs in cancer inflammation and immunity: a leading role for STAT3. Nat Rev Cancer 9: 798-809.

Zhou X, Coad J, Ducatman B, Agazie YM (2008) SHP2 is up-regulated in breast cancer cells and in infiltrating ductal carcinoma of the breast, implying its involvement in breast oncogenesis. Histopathology 53: 389-402.

Zhou X, Agazie YM (2009) Molecular mechanism for SHP2 in promoting HER2-induced signaling and transformation. J Biol Chem 284: 12226-12234.
